# Impact of COVID-19 on liver transplant recipients–A systematic review and meta-analysis

**DOI:** 10.1016/j.eclinm.2021.101025

**Published:** 2021-07-13

**Authors:** Anand V. Kulkarni, Harsh Vardhan Tevethia, Madhumita Premkumar, Juan Pablo Arab, Roberto Candia, Karan Kumar, Pramod Kumar, Mithun Sharma, Padaki Nagaraja Rao, Duvvuru Nageshwar Reddy

**Affiliations:** a Department of Hepatology, Asian Institute of Gastroenterology, Hyderabad, India; b Department of Hepatology, PGIMER, Chandigarh, India; c Departamento de Gastroenterologia, Escuela de Medicina, Pontificia Universidad Catolica de Chile, Santiago, Chile; d Department of Hepatology, Pacific Institute of Medical Sciences, Udaipur, India

**Keywords:** COVID-19, liver transplantation, liver transplant recipients, solid organ transplantation, SARS-CoV-2

## Abstract

**Background:**

Immunosuppression and comorbidities increase the risk of severe coronavirus disease-2019 (COVID-19) in solid organ transplant (SOT) recipients. The outcomes of COVID-19 in liver transplant (LT) recipients remain unclear. We aimed to analyse the outcomes of severe acute respiratory syndrome coronavirus 2 (SARS-CoV-2) infection in LT recipients.

**Methods:**

The electronic databases were searched for articles published from 1 December 2019 to 20 May 2021 with MeSH terms COVID-19, SARS-CoV-2, and liver transplantation. Studies reporting outcomes in more than 10 LT recipients were included for analysis. LT vs non-LT patients with COVID-19 infection were compared for all-cause mortality, which was the primary outcome studied. We also evaluated the relation between the timing of COVID-19 infection post-LT (< one year vs > one year) and mortality.

**Findings:**

Eighteen articles reporting 1,522 COVID-19 infected LT recipients were included for the systematic review. The mean age (standard deviation [SD]) was 60·38 (5·24) years, and 68·5% were men. The mean time (SD) to COVID-19 infection was 5·72 (1·75) years. Based on 17 studies (I^2^ = 7·34) among 1,481 LT recipients, the cumulative incidence of mortality was 17·4% (95% confidence interval [CI], 15·4–19·6). Mortality was comparable between LT (*n* = 610) and non-LT (*n* = 239,704) patients, based on four studies (odds ratio [OR], 0·8 [0·6–1·08]; *P* = 0·14). Additionally, there was no significant difference in mortality between those infected within one year vs after one year of LT (OR, 1·5 [0·63–3·56]; *P* = 0·35). The cumulative incidence of graft dysfunction was 2·3% (1·3–4·1). Nearly 23% (20·71–25) of the LT patients developed severe COVID-19 infection. Before infection, 71% and 49% of patients were on tacrolimus and mycophenolate mofetil, respectively. Immunosuppression was modified in 55·9% (38·1–72·2) patients after COVID-19 infection.

**Interpretation:**

LT and non-LT patients with COVID-19 have a similar risk of adverse outcomes.

**Research in context**

**Evidence before this study**

We searched PubMed from 1 December 2019 to 20 May 2021 with MeSH terms COVID-19, SARS-CoV-2, and liver transplantation. A few systematic reviews, including only 100–200 patients, were published. No major systematic reviews and meta-analyses have described the clinical features, disease course, and outcomes of COVID-19 infected liver transplant (LT) recipients.

**Added value of this study**

To date, this is the largest meta-analysis, including 18 articles and 1522 COVID-19 infected LT patients. The outcomes were compared with 239,704 COVID-19 infected non-LT patients. The cumulative incidence of mortality among LT recipients was 17·4%. Furthermore, LT and non-LT patients had similar mortality (odds ratio, 0·8 [95%CI,0·6–1·08]; *P* = 0·14).

**Implications of all the available evidence**

Currently available evidence suggests that COVID-19 infected LT recipients are not at increased risk of poor outcomes.

Alt-text: Unlabelled box

## 1 Introduction

Coronavirus disease-2019 (COVID-19) is a global pandemic caused by the severe acute respiratory syndrome coronavirus 2 (SARS-CoV-2). The outcomes and severity of COVID-19 are dependent on comorbidities such as diabetes mellitus, cardiovascular diseases including hypertension, kidney disease, pulmonary disease, and age [1], [2], [3]. However, the presence of the underlying liver disease may not impact the outcome of COVID-19 [4]. Solid-organ transplant (SOT), including liver transplant (LT) recipients, being immunosuppressed, are prone to severe infections [5]. Therefore, the presence of comorbidities and chronic immunosuppression may increase the risk of severe COVID-19 among SOT recipients [6,7]. Further, SARS-CoV-2 can worsen liver disease, and LT cannot be delayed due to increased waitlist mortality risk [8,9]. Therefore, transplant centres have initiated SOTs globally [9,10]. Given that the effects of COVID-19 on liver graft injury in LT recipients are unclear, it is crucial to understand the clinical course, management, and outcomes of COVID-19 in these patients [11]. In this systematic review, we reviewed the (a) clinical presentation, management, and outcomes of COVID-19 in LT recipients, (b) immunosuppression at baseline and after the infection, and (c) compared the outcomes of COVID-19 in LT recipients with those who did not undergo LT.

## 2 Methods

We followed the MOOSE (meta-analysis of observational studies in epidemiology) guidelines for data extraction and reporting [12]. The electronic databases were searched from 1 December 2019 to 20 May 2021 with MeSH terms COVID-19, SARS-CoV-2, and liver transplantation. We also searched the abstract books of the International Liver Congress 2020 of the European Association for Study of the Liver (EASL), The Liver Meeting 2020 of the American Association for Study of the Liver Diseases (AASLD), and the Asian Pacific Association for Study of the Liver (APASL) 2021, for data on COVID-19 infection in LT recipients. The reference list of obtained articles and previous meta-analyses were also searched for additional data. Additional articles on COVID-19 and liver transplantation (through 20 May 2021) were identified through the Google search engine. Details of the PubMed search strategy are reported in Appendix I.

### 2.1 Study selection

Studies reporting the outcomes in LT recipients (at least ten or more) were included in the analysis. We excluded case reports, case series (<10 patients), review articles, guidelines, editorials, recommendations, protocols, articles describing paediatric patients, and articles on the number of transplants done during the pandemic.

### 2.2 Data extraction

Data were extracted by two independent investigators (AVK, HVT) and was again cross-checked for accuracy by a third investigator (PK). In case of non-agreement, a senior investigator (PNR) acted as the mediator. From each included study, we recorded the first author, country, age, the number of male patients, time to infection after LT, reason for testing, comorbidities, aetiology of liver disease for which LT was performed, number (%) of patients with each symptom such as fever, cough, dyspnoea, gastrointestinal symptoms, the severity of COVID-19, treatment for COVID-19, mortality, and change in immunosuppressants. We extracted information on baseline inflammatory markers, including ferritin, C-reactive protein (CRP), interleukin (IL)−6, D-dimer, and leucocyte and lymphocyte counts. We noted the number (%) of patients developing thrombotic complications, acute kidney injury (AKI), and secondary bacterial and fungal infections [13]. We also determined the percentage of patients (a) requiring hospitalisation, intensive care unit (ICU) admission, and (b) with abnormal liver enzymes and graft dysfunction. Only articles published in English were included.

### 2.3 Definitions

Patients who tested positive for SARS-CoV-2 (through the nucleic acid test) were considered COVID-19 positive. Patients with symptoms and chest computed tomography (CT) suggestive of classical COVID-19 were considered COVID-19 positive even if the nucleic acid test results were negative. LT recipients were those who underwent transplantation for any indication, such as alcohol-related liver disease, viral (including hepatitis B, hepatitis C), non-alcoholic steatohepatitis (NASH), and hepatocellular carcinoma (HCC). Autoimmune hepatitis (AIH), primary biliary cholangitis (PBC), and primary sclerosing cholangitis (PSC) were grouped as autoimmune liver diseases. The following conditions were considered comorbidities: hypertension, diabetes, cardiovascular (heart failure, coronary artery disease, arrhythmias, stroke, cardiomyopathy), pulmonary (bronchial asthma, chronic obstructive pulmonary disease), and kidney disease. Body mass index > 30 kg/m^2^ indicated obesity. Elevated liver chemistries were characterised by liver enzyme levels (aminotransferases) above the upper limit of normal (or 40 U/L). Mortality due to any cause after COVID-19 infection was considered all-cause mortality. Cause of mortality was noted. Patients requiring non-invasive or invasive mechanical ventilation were considered as being on ventilatory support. Severity was defined as per the Chinese National Health Commission or as reported by the study authors [14]. According to the Health Commission, adult patients meeting any of the following criteria are considered to have a severe infection: respiratory rate ≥30 beats/min in a resting state; mean oxygen saturation ≤93%; arterial blood oxygen partial pressure (PaO2)/oxygen concentration (FiO2) ≤300 mm Hg; CT image showing lesion progression (>50% within 24‐48 hours). In the absence of a definition, we considered patients requiring ventilatory support or patients who succumbed to COVID-19 as having severe COVID-19. AKI was defined as a rise in serum creatinine by 0·3 mg/dl within 48 h from baseline any time after infection or as defined by the study authors [15]. Deep vein thrombosis, acute pulmonary embolism, stroke, or myocardial infarction due to COVID-19 were considered thrombotic events [16]. Biopsy-proven acute cellular and antibody-mediated rejection were considered liver allograft dysfunction. Definitions for graft dysfunction used by study authors were considered if the study did not report biopsy-proven rejection. COVID-19 infection within one year and after one year of an LT was defined as early and late infections, respectively [17].

### 2.4 Outcomes

The primary outcome was all-cause mortality in COVID-19 infected LT recipients. We compared all-cause mortality in infected LT vs non-LT patients. We compared the effects of early vs late infection on mortality. The secondary outcome was the clinical presentation of COVID-19 in LT recipients. Percentages of patients requiring ventilatory support, hospitalisation, and ICU admission were compared between LT vs non-LT patients. We assessed the percentage of patients with graft dysfunction. Lastly, we assessed immunosuppression at baseline (before infection) and the percentage of patients with a change in immunosuppression post-infection.

### 2.5 Study quality

The New-Castle Ottawa scale (NOS) was used to assess the bias in case-control and cohort studies [18]. The NOS tool has three components: cohort selection, comparability, and outcome. The scale can assign a maximum of nine points; four for cohort selection (one each for representativeness of exposed cohort, selection of non-exposed cohort, ascertainment of exposure and demonstration that the outcome of interest was not present at the start of the study), two for comparability and three for outcome assessment (one each for assessment of outcome, sufficient duration of follow-up and adequacy of follow-up). Any discrepancy in the study quality assessment was discussed, and the best score was decided after confirming with a third investigator (PNR) who acted as the mediator.

### 2.6 Statistical analysis

Data retrieved were entered in Microsoft Excel. Baseline data, including gender, liver disease aetiology, comorbidities, and other categorical data are expressed as n (%). Continuous data (e.g., age, time to infection, inflammatory markers) are expressed as mean (standard deviation [SD]). Median and interquartile range (IQR) were converted to mean and SD to summarise the results. Baseline data were analysed using SPSS ver. 25 (IBM Corp, Armonk, NY, USA). Meta-analysis was performed using Comprehensive Meta-analysis software (ver. 3.3. 2014, USA). The event rate of each outcome (mortality, ventilatory support, hospitalisation, graft dysfunction, and immunosuppression) was expressed as a percentage. In the absence of reported percentages, we re-calculated percentages to summarise results. We transformed pooled data through logit transformation to assess the pooled incidence. The sample size (n) was entered, and the analysis was run. Studies with NOS scores <7 were excluded from sensitivity analyses. For studies reporting proportions, odds ratios were used to describe the differences between LT and non-LT patients. Meta-analysis was performed using random effect models. Forest plots were generated to present the pooled estimates. For 0-values, the software is programmed to correct readings due to the imposition of standard (0.5) continuity correction in forest plots. We assessed heterogeneity using I^2^ statistics and Q measures. *P* values <0·1 were considered statistically significant. Publication bias was assessed quantitatively (Egger's regression; intercept test) and qualitatively (visual examination of funnel plot symmetry) [19].

### 2.7 Role of the funding source

There was no funding source for this study.

## 3 Results

**Fig. 1:**
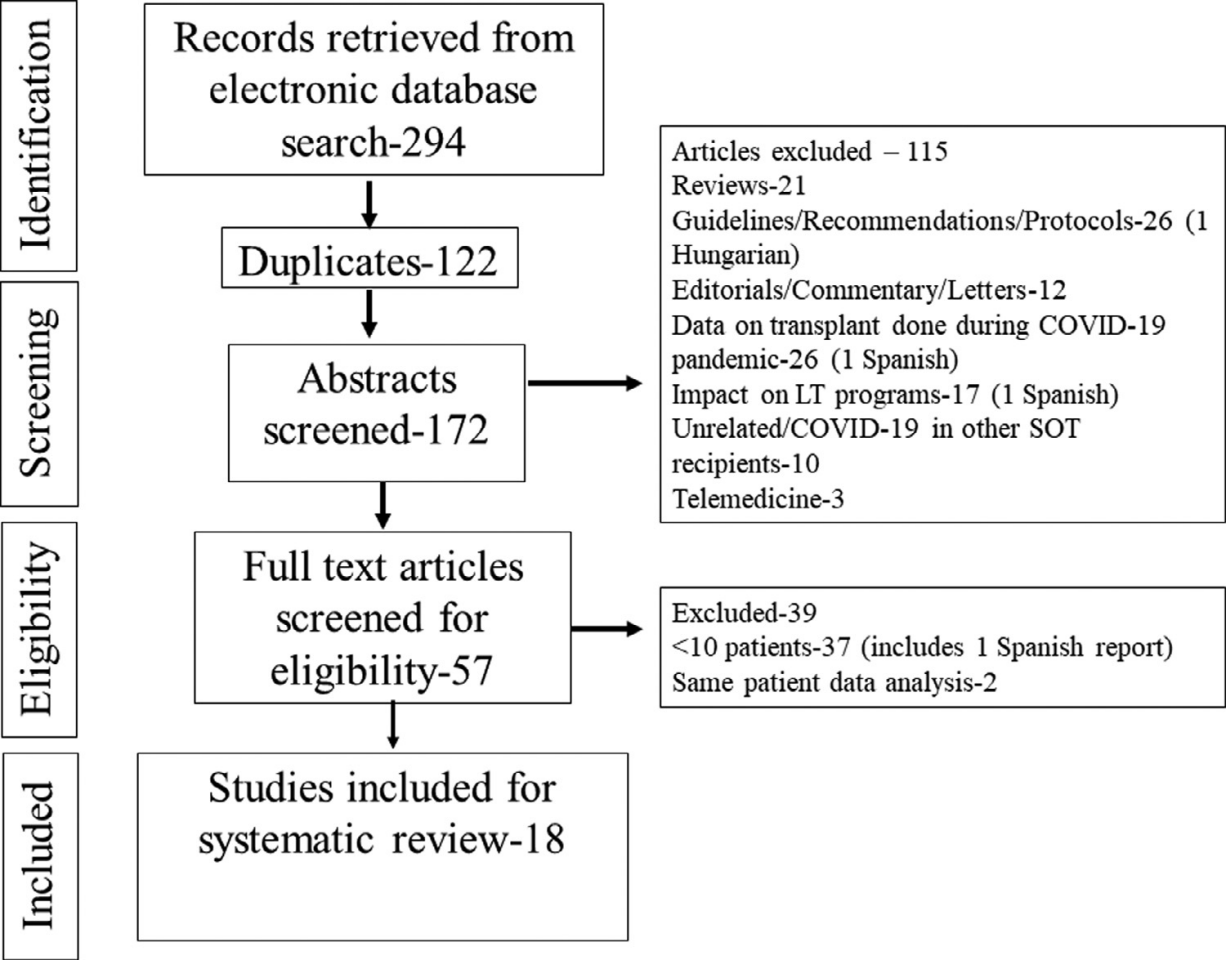
Flowchart depicting the articles retrieved, excluded, and included in the study.

We retrieved 294 articles through the database search. After screening 172 abstracts (excluding 122 duplicates), 57 full-text articles were reviewed (Fig. 1). Thirty-seven articles reporting <10 patients and two articles reporting the preliminary data for the same patients were excluded [20,21]. Therefore, a total of 18 articles reporting 1522 patients were included for the systematic review and meta-analysis. [16,17,[22], [23], [24], [25], [26], [27], [28], [29], [30], [31], [32], [33], [34], [35], [36], [37]] (Supplementary file 2: Excluded case reports and other language articles).

### 3.1 Characteristics of liver transplant recipients and controls

Of the patients evaluated, 68·5% (770/1124) were men, with a mean age (SD) of 60·38 (5·24) years. Gender and age were not reported in four studies [31], [32], [33], [34]. All patients had positive polymerase chain reaction (PCR) tests, except five studies including patients with classical symptoms and CT features suggestive of COVID-19 (Appendix II) [[25], [26], [27],^32^,^37^]. Only 14 asymptomatic patients were detected to be PCR positive when screened for hospital admission or prior to a planned procedure (or surgery) or after a contact exposure [22,23,28,35]. The aetiology of liver disease was reported in nine studies and 855 LT recipients. [17,[22], [23], [24], [25],^27^,^29^,^30^,^37^] The most common aetiology was viral (38%, 318/855), followed by alcohol-related liver disease (22·23%, 190/855), NASH (8·5%, 72/855), autoimmune liver disease (7·4%, 63/855), and HCC (5·26%, 45/855). Other causes contributed to 18·5% (158/855) of aetiologies, and lastly, 8·2% (70/855) of patients had concomitant HCC.

Except for six articles, the other studies reported comorbidities in 1095 patients. [26,27,[31], [32], [33], [34],^37^] The most common comorbidities were hypertension (44·3%, 485/1095), diabetes (39·4%, 431/1095), cardiovascular (16·43%, 180/1095), kidney (13·7%, 150/1095), and pulmonary disease (9%, 99/1095). Three articles reported angiotensin-converting enzyme inhibitor (ACEi) use in 27% (70/259) of patients. [17,22,37] Obesity was reported in seven articles, and 23·6% (158/670) of LT recipients were obese (BMI > 30 kg/m^2^) [17,23,24,26,29,30,36]. Table 1 describes the baseline demographics of the included patients.

**Table 1:** Demographics of the liver transplant recipients.

Serial no	First author, Country, Centres	Number of liver transplant patients	Age in years (median and IQR)	Males (n,%)	Aetiology of liver disease	Comorbidities, n (%)
1	Colmenero et al., Spain, Multicentre [22]	111	65·34±10·96ǂ	79/111 (71·2%)	ARLD-34 (30·6%) Viral-44 (39·63%) Autoimmune liver disease- 9 (8·1%)	HTN-64 (57·7%) DM- 53 (47·7%) ACEi-33 (29·7%) Cardiovascular-22 (19·8%) Bronchopulmonary-13 (11·7%)
2	Becchetti et al., Switzerland, Multicentre [17]	57	65 (57–70)	40 (70%)	ARLD-15(26·31%) Viral-24 (42%) Autoimmune liver disease-8 (14%) NAFLD-2 (4%) HCC-20 (35%) Other-12 (21%)	HTN-32 (56%) DM-21 (37%) Cardiovascular-21 (37%) Malignancy-5 (9%) Pulmonary disease-13 (23%) Kidney disease-16 (28%) ACEi-13 (23%) Obesity −8 (14%)
3	Lee et al., USA, Single centre [23]	38	63 (27–81)	26/38 (68%)	ARLD-2 (5%) Viral-18 (47%) NAFLD-6 (16%) HCC-8 (21%) Autoimmune liver disease-8 (21%) Other-4 (10·58%)	HTN-24/38 (63%) DM-18/38 (47%) Cardiovascular-11/38 (29%) CKD-24/38 (63%) Malignancy- 2/38 (5%) Obesity-10/24 (42%)
4	Webb et al., UK, Multicentre [24]	151	60 (47–66) vs 73 (55–84)	102/151 (74%) vs 329/627 (52%)	ARLD-19 (13%) Viral- 40 (26·5%) NASH-20 (13%) Autoimmune liver diseases- 19 (13%)	HTN-63 (42%) DM-65 (43%) Pulmonary −8 (6%) Cardiac-22 (15%) Obesity (BMI>30) −44 (29%)
5	Malekhosseini. et al. Iran, Single centre [25]	66 LT (4 paediatric)	45·9 ± 16·7ǂ	52 (78·8)	ARLD-1 (1·5%) Viral-16 (24·3%) NASH-10 (15·2%) Autoimmune liver diseases-19 (29%) Other-21 (30%)	HTN-7 (10·6%) DM −16 (24·2%) Pulmonary disease-1 (1·5%) Cancer-1 (1·5%) Cardiovascular-2 (2·4%)
6	Patrono et al., Italy, Single centre [26]	10	65·6	8/10(80%)		Obesity 1/10 (10%)
7	Loinaz et al., Italy, Single centre [27]	19 (1SLKT) 17 RT-PCR and 2 classical CT and symptoms	58 (55–72)	Males-14/19(73·6%)	ARLD-3 Viral-13 Cryptogenic-1 ALF-2 Concomitant HCC-7	Obesity 10/19 (52·4%)
8	Dhampalwar et al., India, Single centre [28]	12	53·6 ± 9·2 yearsǂ	11/12(91·6%)		HTN-4/12 (33/3%) DM-9 (75%)
9	Rabiee et al., USA, Multicentre [29]	112	61	61//112 (54·5%)	ARLD-16 (14·3%) Viral-40 (36%) NAFLD-16 (14·3%) HCC-17 (15·2%) Others-23 (20·53%)	HTN-59/112 (53·2%) DM-51/112 (45·5%) Pulmonary disease-11 (10%) Cardiovascular-15 (13·4%) Cancer-7 (6·3%) Obesity-26 (23·4%) Hyperlipidemia-23 (20·7%)
10	Mansoor et al., USA, Multicentre [16]	126	57·08 ± 13·28ǂ	83 (66%)		HTN-29 (23%) DM-20 (16%) Cardiovascular-20 (16%) Pulmonary disease-10 (8%) CKD-25 (20%)
11	Belli et al., Europe, Multicentre. [30]	243	63 ǂ	171 (70·37)	ARLD- 60 (24·69%) NAFLD/NASH-18 (7·41%) Viral-105 (43·2%) Other-79 (32·51%) Missing-2 (0·82%) (Concomitant) HCC-63/243 (26%)	Hypertension-111 (45·68%) Diabetes-94 (38·68%) Coronary artery disease-17 (7%) Pulmonary disease-25 (10·29%) Chronic kidney disease-49 (20·16%) Other-43 (17·70%) None-57 (23·46%) Obesity 46 (19%)
12	Trapani et al., Italy, Multicentre [31]	89 liver transplants				
13	Polak et al. Netherlands, Multicentre [32]	244/272 symptomatic	62 ± 14	162 (67%)		
14	Gruttadauria et al., Italy, Multicentre [33]	24				
15	Pereira et al., USA, Multicentre [34]	13				
16	Ali et al., Saudi Arabia, Single centre [35]	15	62·7 (14·9)ǂ	11 (73·3%)		Diabetes-10 (66·7%) HTN-2 (13·3%) IHD-1 (6·7%)
17	Kates et al., USA, Multicentre [36]	73	62 (50–67)	48 (65·8%)		DM-34 (46·6%) HTN-39 (53·4%) Cardiovascular-17 (23·3%) Pulmonary-5 (7%) Kidney-36 (49·3%) Obesity-23 (31·5%) Malignancy-3 (4·1%)
18	Dumortier et al. France, Multicentre [37]	91 adult patients (Liver kidney-12 and Liver-heart-1)	64·4 (54·9–71·3)	64 (70·3%)	ARLD-40/91 Viral-18/91 Not reported-23/91	HTN-51 (56%) Diabetes-40 (44%) Cardiovascular disease- 32 (35·16%) Respiratory disease-13 (14·3%) Overweight (BMI>25)−44 (62%) ACEi-24 (26·4%)

ǂmean (standard deviation) ARLD-alcohol-related liver disease; NAFLD-non-alcoholic fatty liver disease; HCC-hepatocellular carcinoma; HTN-hypertension; DM-diabetes Mellitus; ACEi-angiotensin-converting enzyme inhibitor; BMI-body mass index; CKD-chronic kidney disease; IHD-ischemic heart disease; Other causes of liver disease include- metabolic liver diseases, polycystic liver disease, drug-induced liver injury, or Budd-Chari syndrome.

Five articles compared the outcomes in LT and non-LT patients (*n* = 240,079) [16,24,29,31,32]. Webb et al. included a control group of 627 COVID-19 patients, of whom 1% had cirrhosis and 0·5% received other organ transplants [24]. Rabiee et al. included COVID-19 infected non-transplant chronic liver disease (CLD) patients (*n* = 375) as controls [29]. Mansoor et al. included 125 non-LT patients matched for age, race, and key comorbidities, and Trapani et al. included 238,895 non-SOT COVID-19 infected patients [16,31]. Polak et al. included 57 LT candidates [32]. While 438 patients were CLD patients, only three were other SOT recipients. The aetiology of CLD was not reported, and 45·5% (109,219 / 240,060) of the patients were men. The mean age (SD) was 62·56 (5·25) years. Three studies reported comorbidities [16,24,29]. Supplementary Table 1 describes the baseline characteristics of control patients included in the systematic review. Common comorbidities included hypertension (43%, 486/1127), cardiovascular disease (40·55%, 305/752), diabetes (30·2%, 341/1127), pulmonary disease (22·6%, 170/752) and kidney disease (16%, 121/752). Obesity was reported in 33·43% (335/1002) of patients. A higher percentage of non-LT patients had obesity, cardiovascular and pulmonary diseases, while diabetes was more frequent in LT recipients. Hypertension and kidney disease were similar in both groups (Supplementary Table 2a). Two studies reported immunosuppressant use in non-LT patients. Five percent (37/752) of patients were on steroids; 2.2% were on calcineurin inhibitors (CNIs) (16/752) and anti-metabolites (17/752).

### 3.2 COVID-19 infection in LT recipients

Twelve studies reported COVID-19 symptoms among 994 LT recipients [16,17,[22], [23], [24], [25], [26], [27], [28],^30^,^36^,^37^]. Fever was the most common symptom (49·7% [46·5–52·85], 494/994), followed by cough (43·76% [40·65–47], 435/994), and dyspnoea (29·27% [26·46–32·21], 291/994). Gastrointestinal (GI) symptoms were noted in 27·26% (24·51–30·14, 271/994) patients (Table 2). Based on 12 studies, the mean (SD) time to COVID-19 infection in 1065 patients was 5·72 (1·75) years. Inflammatory markers were reported among eight studies [16,17,22,23,25,30,35,37]. The mean (SD) values of ferritin, IL-6, CRP, and D-dimer were 731·25 (346·87) ng/mL, 48·95 (24·53) pg/mL, 74·22 (30·74) mg/L, and 1092·75 (463·33) ng/mL, respectively (Table 3).

**Table 2:** Symptoms of COVID-19 in liver transplant recipients.

Symptoms	% (95%CI)	n/N

Fever	49·7% (46·5–52·85)	494/994
Cough	43·76% (40·65–47)	435/994
Dyspnoea	29·27% (26·46–32·21)	291/994
Gastrointestinal symptoms	27·26% (24·51–30·14)	271/994
Myalgia	18% (15·67–20·54)	179/994
Fatigue	11·26% (9·36–13·39)	112/994
Neurological symptoms (confusion, headache)	6·43% (5–8·14)	64/994
Anosmia	3.72% (2·63–5·1)	37/994
Rhinorrhoea	3·42% (2·38–4·47)	34/994
Sore throat	2·81% (1·87–4·04)	28/994
Anorexia	2·21% (1·4–3·34)	22/994

**Table 3:** Baseline inflammatory markers in COVID-19 infected liver transplant recipients.

Markers	Mean (SD)	N	References

Ferritin (ng/ml)¶^,^ǁ	731·25 (346·87)	163	[17,22,23,35]
Interleukin-6 (pg/ml) ǂ	48·95 (24·53)	149	[16,23]
C-reactive protein (mg/L)¶	74·22 (30·74)	151	[23,25,35,37]
D-dimer (ng/ml)¶	1092·75 (463·33)	167	[17,22,23,35]
Total leucocyte count (cells/µL)	5259 (927·47)	485	[16,17,25,30]
Lymphocytes (cells/µL)	710 (81·54)	347	[16,17,22,23,37]

¶ Study by Mansoor et al. excluded due to abnormal value/units (outlier).

ǂ Study by Becchetti et al. excluded due to lack of abnormal value/units (outlier).

ǁ Colemenro et al. reported maximum ferritin levels.

COVID-19 infection was severe in 22·8% (95%CI, 20·71–25, 347/1522) of infected patients. Most studies defined severe disease as patients requiring ventilatory support or mortality due to COVID-19 (Appendix II). The cumulative incidence of AKI was 33·22% (95% CI, 28·02– 38·74, 104/313) among six studies that reported it. [[23], [24], [25],^28^,^35^,^37^] Cumulative incidence of stage three AKI or requirement of renal replacement therapy was 55·22% (95% CI, 50·44–60, 243/440) reported among three studies. [24,30,37] Seven studies reported thrombotic complications among 5·75% (95%CI, 3·94–8·06, 31/539) of LT patients [16,23,27,30,34,36,37]. Secondary bacterial infections were reported among 11·6% (95%CI, 8·65–15·13, 47/405) and fungal infections were reported among 2·58% (95%CI, 1·18–4·86, 9/348) of LT recipients among four studies [17,23,30,37].

COVID-19 therapies included hydroxychloroquine (HCQ), antibiotics (azithromycin, imipenem, cotrimoxazole, vancomycin), antivirals (lopinavir/ritonavir (Lpv/r), remdesivir, oseltamivir, sofosbuvir, darunavir/cobicistat), immunomodulators (tocilizumab, rituximab, ruxolitinib, interferon, anakinra), steroids (irrespective of the type and mode of administration), anticoagulants (low molecular weight or unfractionated heparin), and other medications (fluconazole, guanfacine).

Eleven studies reported therapies administered to 852 COVID-19 infected LT recipients. The most prescribed drug were HCQs (48·23%, 411/852), followed by antibiotics (36·15%, 308/852), antivirals (14·2%, 121/852), immunomodulators (5·63%, 48/852), steroids (7·62%, 65/852), and other drugs (3·52%, 30/852). Anticoagulation was reported in three studies, and 30% (126/418) of patients received it [23,24,30]. One study reported the use of plasma therapy for two patients [24]. Supplementary Table 3 describes the characteristics of COVID-19 in each study.

### 3.3 Mortality in LT recipients infected with COVID-19

**Fig. 2:**
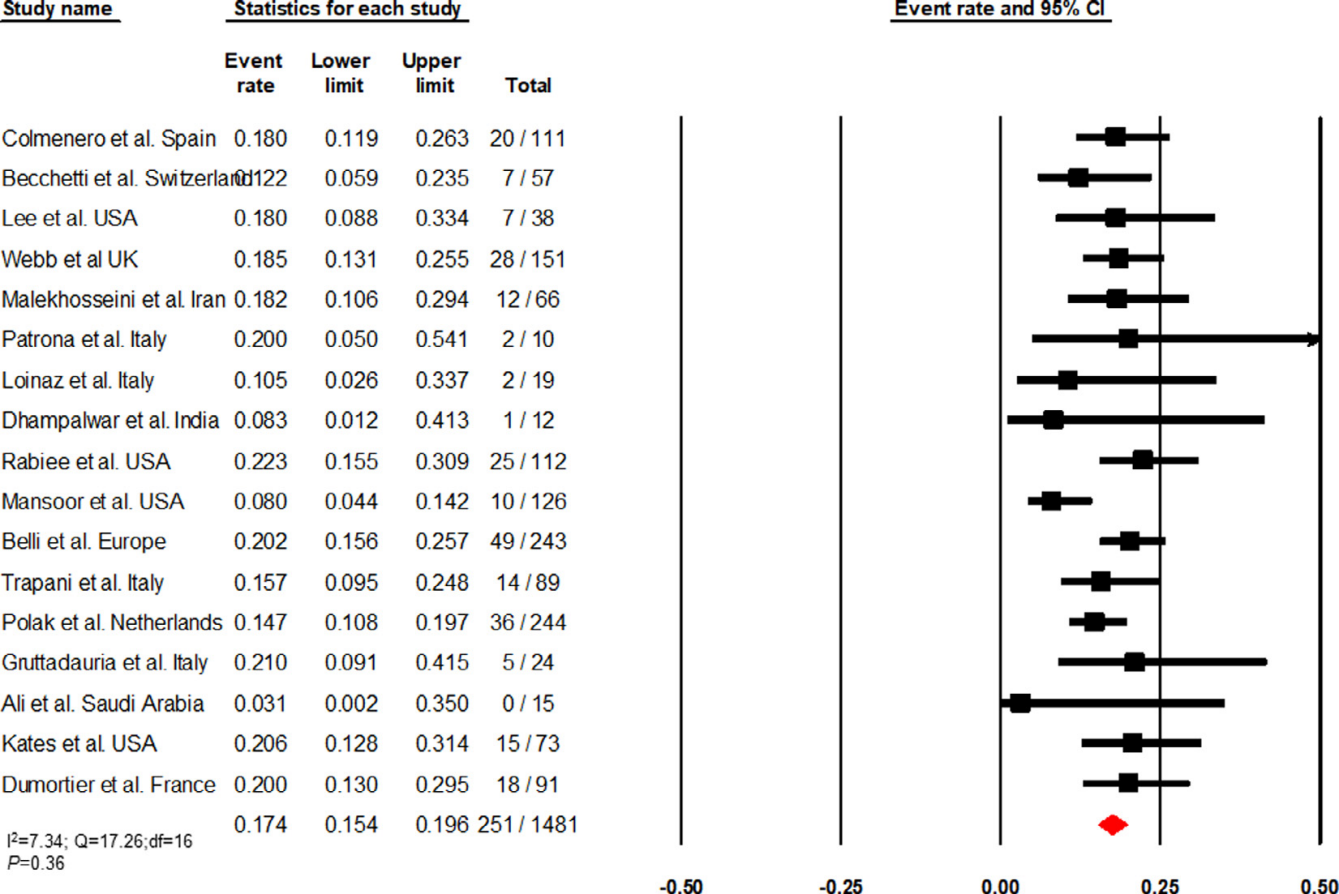
Forest plot depicting the cumulative incidence of mortality in COVID-19 infected liver transplant recipients.

Based on 17 articles, the cumulative incidence of mortality among COVID-19 infected LT recipients was 17·4% (95% CI, 15·4–19·6, 251/1481) (I^2^=7·34) **(**Fig. 2**)**. Sensitivity analysis of 11 studies with a NOS score ≥ 7 (I^2^ = 34·66) revealed a mortality of 16·5% (95% CI, 14–19·5, 203/1233) (Supplementary Fig. 1). Causes of death were reported as COVID-19 related complications in 62·54% (95% CI, 56·24–68·55, 157/251), pulmonary failure in 29·88% (95% CI, 24·28–36, 75/251), liver-related in 1·6% (95% CI, 0·43–4·02, 4/251), cardiogenic in 0·8% (95% CI, 0·1 – 2·84, 2/251), and other causes in 5·17% (95% CI, 2·78– 8·7, 13/251).

**Fig. 3:**
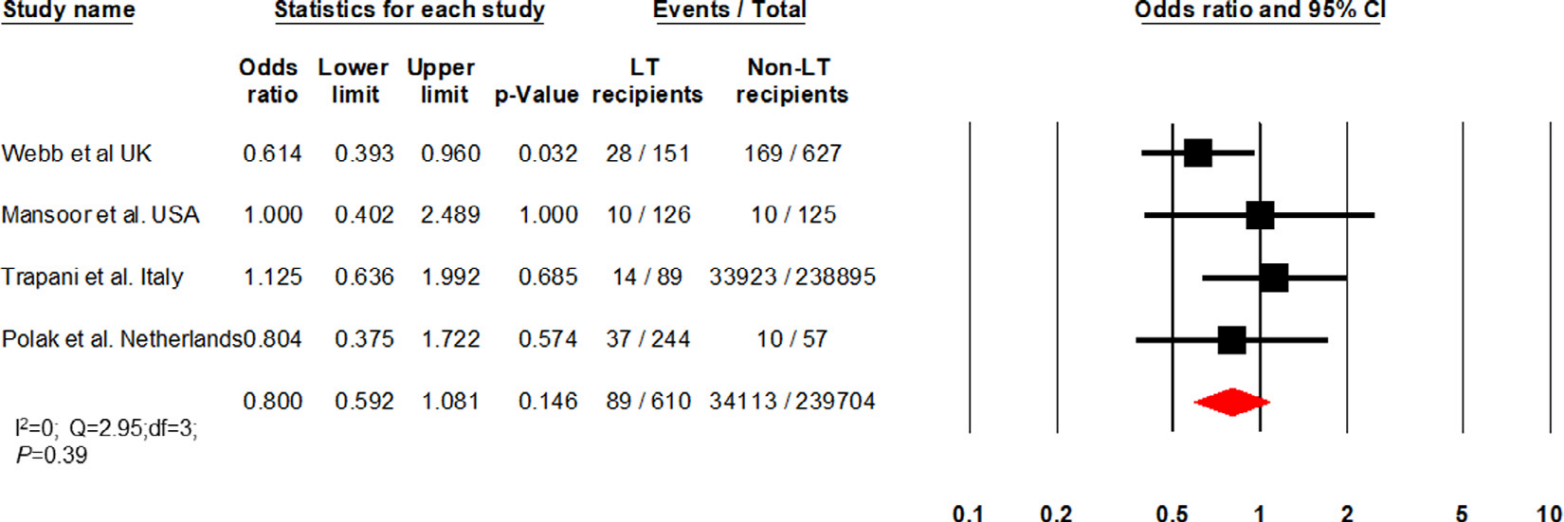
Forest plot comparing mortality between liver transplant (LT) recipients and non-LT patients.

Four articles (I^2^ = 0) comparing LT vs non-LT patients found comparable mortality between groups (OR: 0·8 [0·6 – 1·08]; *P* = 0·14; 89/610 vs 34,113/239,704) (Fig. 3) [16,24,31,32]. The mean ages (SD) of LT and non-LT patients were 59·55 (2·05) and 63·2 (6·57) years, respectively (*P* = 0·91). In the comparison cohort, 63 patients had CLD, and three were other SOT recipients. Hypertension, kidney disease, and obesity were similar in both groups. Cardiovascular and pulmonary diseases were more common in non-LT patients, while diabetes was more common in LT recipients (Supplementary Table 2b).

### 3.4 Effect of timing of COVID-19 infection after transplant on mortality

**Fig. 4:**
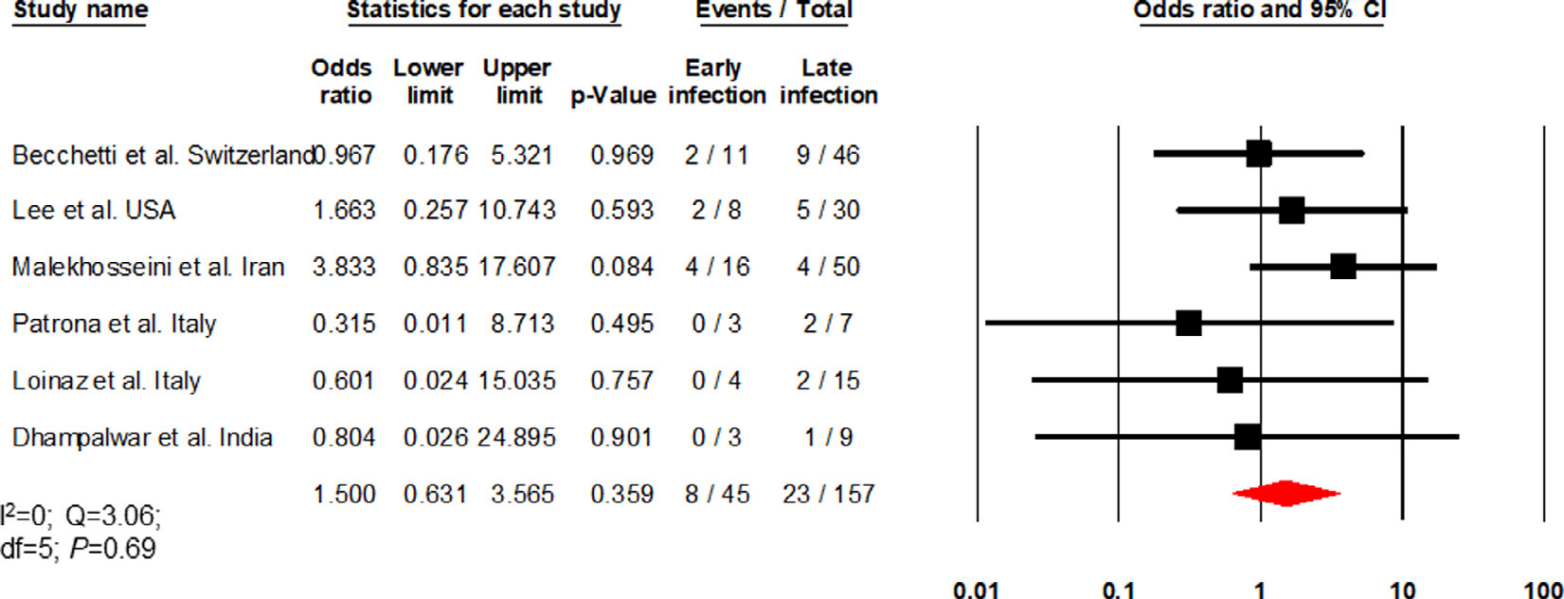
Forest plot comparing mortality among COVID-19 infected liver transplant recipients based on the timing of infection.

Six articles (I^2^=0) reported mortality based on time to infection after LT. There was no difference in mortality between those infected within one year vs after one year (OR, 1·5 [0·63–3·56]; *P* = 0·35; 8/45 vs 23/157) (Fig. 4). A sensitivity analysis of three studies (I^2^=0) with NOS scores ≥ 7 showed that the timing COVID-19 infection did not affect mortality (OR, 1·81 [0·62–5·29]; *P* = 0·27; 6/31 vs 15/111) (Supplementary Fig. 2).

### 3.5 Hospitalisation and ICU care

**Fig. 5:**
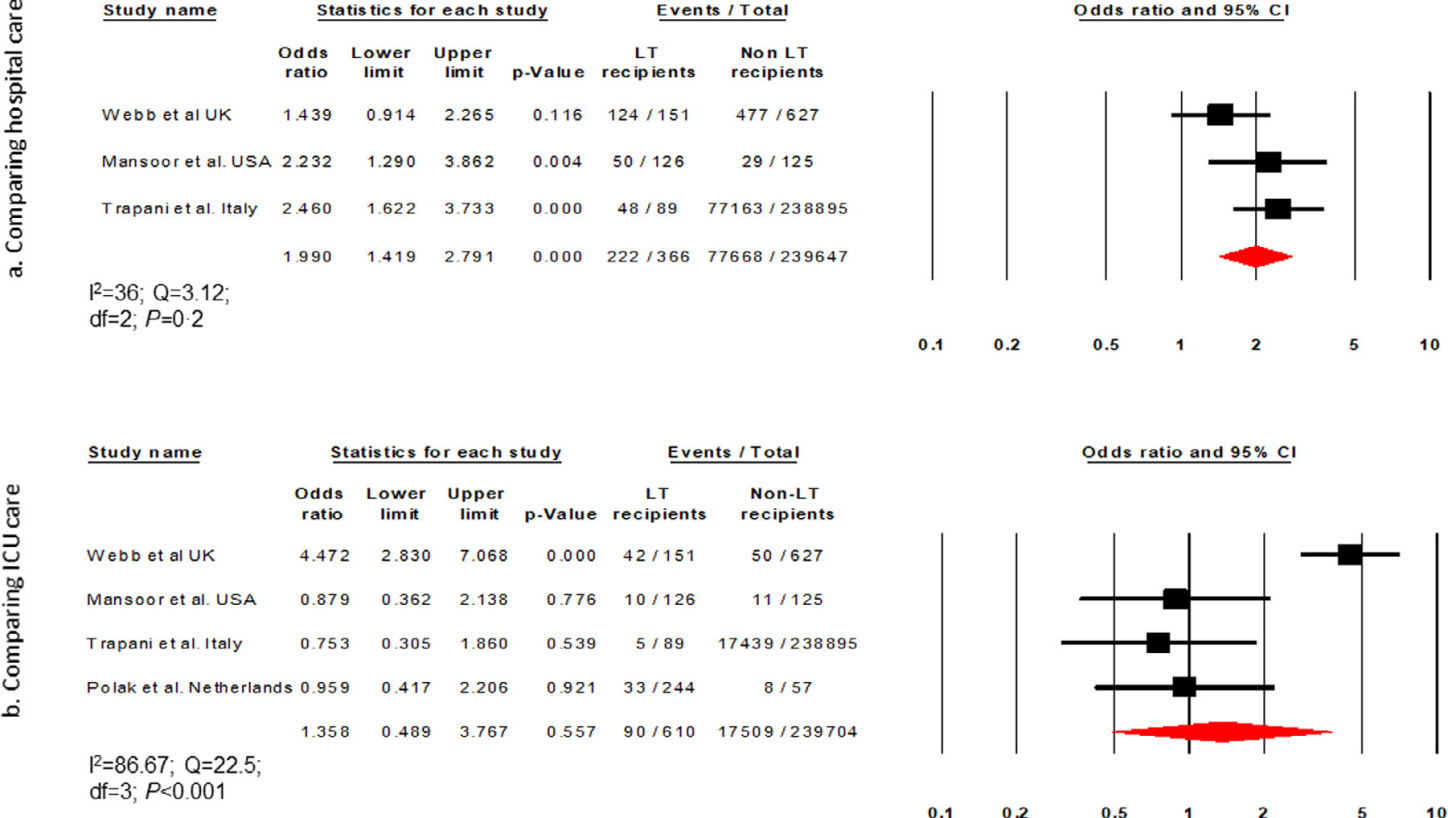
Forest plot comparing liver transplant (LT) and non-LT patients for (a) hospitalisation and (b) intensive care unit admission.

Based on 14 articles (I^2^=87·77) the cumulative incidence of hospitalisation was 72% (63–79·6, 836/1159) among COVID-19 infected LT recipients (Supplementary Fig. 3). Three studies (I^2^ = 36) compared hospitalisation in LT vs non-LT patients [16,24,31]. Hospitalisation was higher in LT than non-LT patients (OR, 1·99 [1·41–2·8]; *P* < 0·001; 222/366 vs 77,668/239,647) (Fig. 5a).

Fifteen studies (I^2^=75·5) reported the cumulative incidence of ICU admission to be 16% (12·1–20·9, 230/1380) (Supplementary Fig. 4). Four articles (I^2^=86·67) comparing ICU admission between LT and non-LT patients reported similar rates of ICU care (OR, 1·35 [0·48–3·76]; *P* = 0·55; 90/610 vs 17,509/239,704) (Fig. 5b).

### 3.6 Ventilatory support

Thirteen articles (I^2^=0) reported that 21·1% (18·6–23·8, 201/974) of patients needed mechanical ventilation (Supplementary Fig. 5). Only one article compared the requirement for ventilatory support between LT and non-LT patients and found that the former required it more often than the latter (19·87% vs 5·1%; *P* < 0·011; 30/151 vs 32/627) [24]. However, this study reported a similar hospitalisation rate but lower mortality among LT recipients [24].

### 3.7 Graft dysfunction

**Fig. 6:**
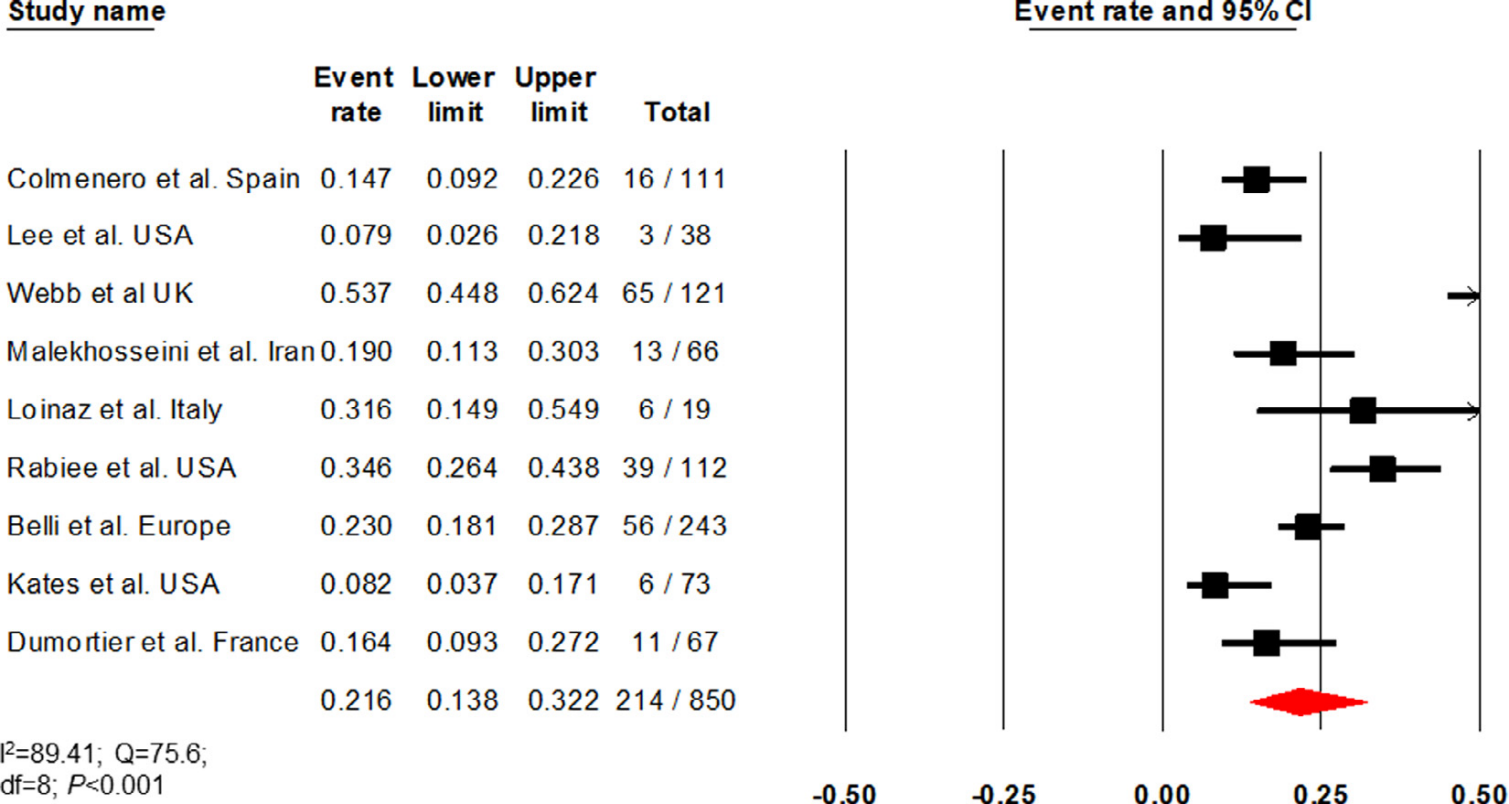
Forest plot depicting the cumulative incidence of elevated liver chemistries.

**Fig. 7:**
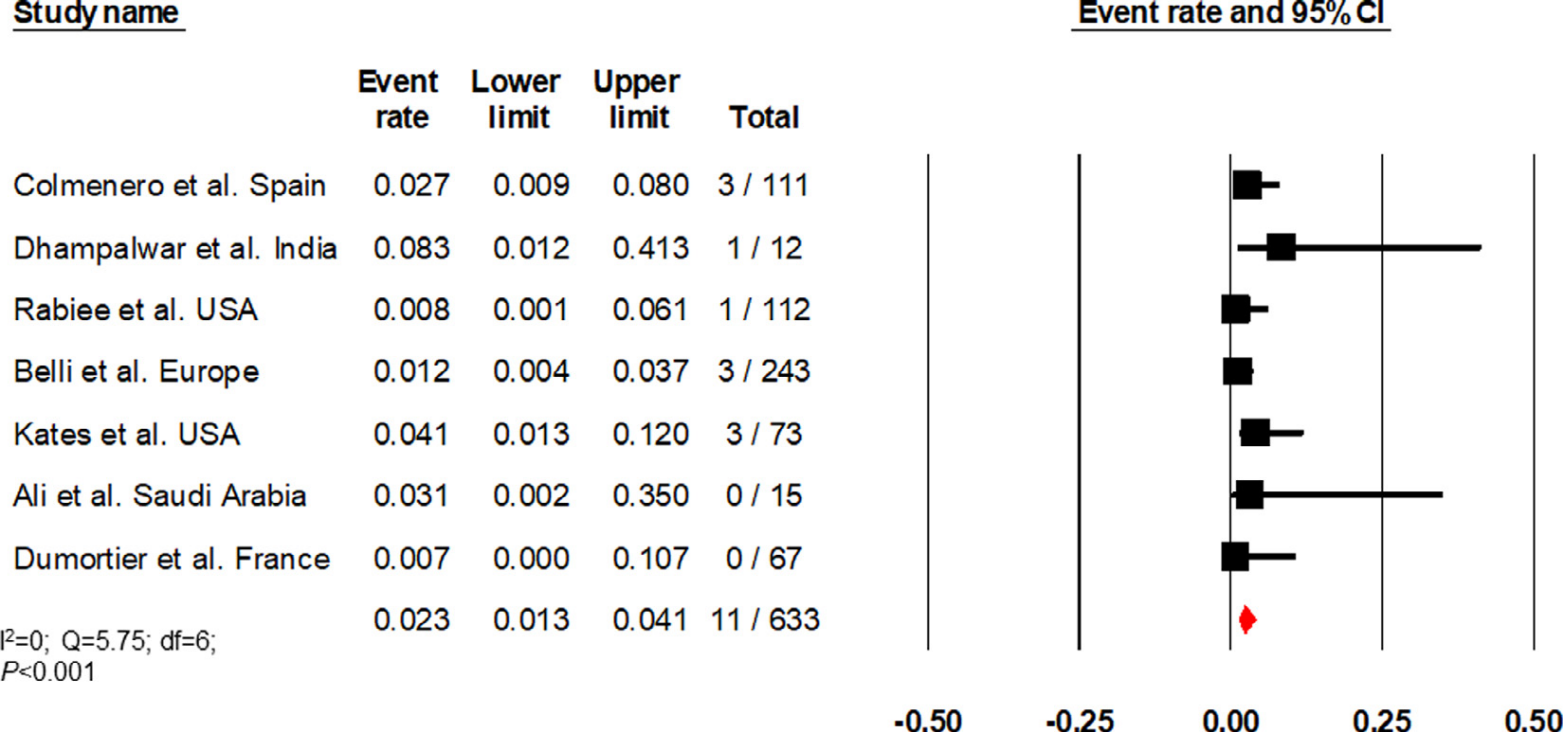
Forest plot depicting the cumulative incidence of graft dysfunction.

Nine studies (I^2^=89·41) reported elevated liver chemistries in 21·6% (13·8–32·2, 214/850) of patients (Fig. 6) [[22], [23], [24], [25],^27^,^29^,^30^,^36^,^37^]. Two studies (I^2^=87·06) that compared elevated liver chemistries between LT and non-LT patients found no difference (OR, 0·9 [0·4–2·05] *P* = 0·79; 104/233 vs 384/823) (Supplementary Fig. 6) [24,29]. Studies by Rabiee et al., Belli et al., Kates et al., and Dumortier et al. reported biopsy-proven acute cellular/antibody rejection [29,30,36,37]. Colmenero et al. defined graft dysfunction as *a* >4-fold rise in bilirubin from baseline or international normalised ratio >1·4 [22]. Dhampalwar et al. and Ali et al. did not define graft dysfunction [28,35]. Based on these seven studies (I^2^=0), the cumulative incidence of graft dysfunction was 2·3% (1·3–4·1, 11/633) among LT recipients (Fig. 7). [22,[28], [29], [30]]

### 3.8 Immunosuppression in LT recipients

Fourteen articles reported baseline (before infection) immunosuppression in 1121 patients (Supplementary Table 4). Most patients were on tacrolimus or mycophenolate before infection (Table 4).

**Table 4:** Baseline immunosuppression in liver transplant recipients.

Immunosuppressant	% (95%CI)	n/N

Tacrolimus	70·82% (67·97–73·56)	743/1049
Mycophenolate	48·84% (44·76–51·93)	507/1038
Steroids	31·91% (28·97–34·96)	307/962
mTORi	11·73% (9·9–13·78)	130/1108
Cyclosporin	7·45% (5·9–9·2)	75/1006
Azathioprine	3·81% (2·31–5·9)	19/498
CNI+antimetabolite	30% (22·9–37·28)	50/168
CNI+steroids	11·53% (6·6–18·31)	15/130
CNI+mTORi	23·8% (17·58–31)	40/168
CNI+antimetabolite+steroids	31·5% (21·13–43·44)	23/73

mTORi- mechanistic target of rapamycin inhibitor; CNI-calcineurin inhibitor.

Eight articles (I^2^=94·1) reported a change in immunosuppression in 55·9% (38·1–72·2, 449/776) of infected LT recipients (Supplementary Fig. 7). Detailed changes in immunosuppression were provided for 56% (252/449) of patients. Calcineurin inhibitors (CNIs) was modified in 49·6% (125/252) (reduced in 56% [70/125], and stopped in 44% [55/125]); mycophenolate mofetil (MMF) (anti-metabolites) was modified in 42·06% (106/252) (stopped in 86% [91/106], and reduced in 14% [15/106]) patients; mTOR (mechanistic target of rapamycin) inhibitors were stopped in 6·74% (17/252); and steroids were reduced in 0·8% (2/252). MMF was associated with a higher risk of mortality in one study [22]. However, other studies reported no effects of baseline immunosuppression and changes in immunosuppression (including complete withdrawal) on liver injury, disease severity, or mortality [23,24,29,30]. Tacrolimus was reported to prevent mortality on multivariate analysis [30].

### 3.9 Study quality

**Fig. 8:**
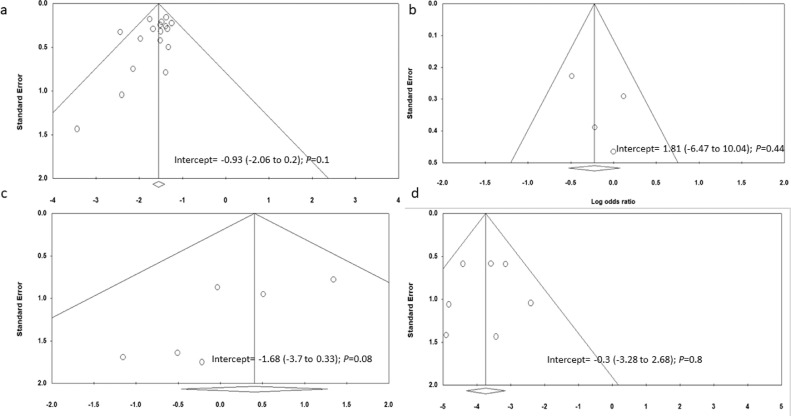
Funnel plot of studies on (a) the incidence of mortality, (b) comparison of mortality between liver transplant and non-solid organ transplant recipients, (c) mortality in COVID-19 infected liver transplant recipients based on the timing of infection, and (d) incidence of graft dysfunction.

Based on funnel plot symmetry and Egger's intercept test, no publication bias was seen in 17 studies reporting mortality (intercept = −0·93; −2·06 to 0·2; *P* = 0·1) (Fig. 8a). Studies comparing mortality between LT and non-LT patients had symmetrical funnel plots (Egger's test) suggestive of no publication bias (intercept = 1·81; −6·47 to 10·04; *P* = 0·44) (Fig. 8b). We found publication bias in six studies reporting mortality based on timing of infection (intercept = −1·68; −3·7 to 0·33; *P* = 0·08) though there was no heterogeneity (Fig. 8c). Studies reporting graft dysfunction had no publication bias (intercept = −0·3;−3·28 to 2·68; *P* = 0·8) (Fig. 8d). No publication bias was seen in studies reporting hospitalisation (intercept = 1·1; −3·56 to 5·78; *P* = 0·61), ICU admission (intercept = −1·71; −4·15 to 0·73; *P* = 0·15), and elevated liver chemistries (intercept = −3·8; −9·38 to 1·77; *P* = 0·15) despite the significant heterogeneity in these studies (Supplementary Fig. 8a-c). On the contrary, studies reporting the need for ventilatory support had low heterogeneity but showed publication bias (intercept = −1·01; −1·96 to −0·07; *P* = 0·03) (Supplementary Fig. 8d). Five, ten and three studies had NOS scores of 8–9, 6–7, and 5, respectively (Supplementary Table 5).

## 4 Discussion

This meta-analysis demonstrated (a) comparable mortality (17·4%) in LT and non-LT COVID-19 patients, (b) 23% of infected patients develop severe disease, (c) hypertension, diabetes, and obesity were the common comorbidities in infected patients, (d) while 72% of patients were hospitalised, only 16% required ICU care, (e) while more LT recipients required hospitalisation than non-LT patients (OR, 1·99 [1·41–2·8] *P* < 0·001) the requirement for ICU care was comparable in both groups and (f) cumulative incidence of graft dysfunction was 2·3% (1·3–4·1). Most of the patients were on tacrolimus or MMF before infection. The most commonly modified immunosuppressant was CNI, followed by MMF.

Age > 60 years is a risk factor for COVID-19-related mortality [38]. In our analysis, mean age and mortality rate were comparable in LT and non-LT patients. In contrast, comorbidities were unevenly distributed (diabetes was more common in LT recipients). Despite a higher proportion of patients with cardiovascular and pulmonary disease among non-LT patients, mortality was similar in both groups. The reported mortality in COVID-19 infected cirrhosis patients ranges between 30% and 42% [39], [40], [41], [42]. The mortality rate for LT recipients (17·4%) was lower than that for other organ transplant recipients [43,44]. LT recipients may not be at higher risk for severe COVID-19 infection. The timing of infection after LT did not affect mortality.

The rate of hospitalisation was high, probably as a precautionary measure in immunosuppressed LT recipients. However, the need for ICU care, ventilatory support, and severe COVID-19 infection were comparable to the general population [45].

The mean time from LT to infection was 5·7 years. Since the number of patients undergoing LT had substantially decreased during the COVID-19 pandemic, it may be premature to conclude that these patients are not prone to infection in the post-operative period. Furthermore, LT recipients have been exposed to COVID-19 for the past 16–18 months. The mean values (time to infection) reported are the average time from transplant to infection in just those LT patients who have already contracted COVID-19. Many more LT patients may be infected later as the number at risk increases with time. LT patients should be screened regularly and monitored for symptoms of COVID-19, including fever and gastrointestinal symptoms.

Unlike kidney transplant recipients, LT recipients are not prone to graft dysfunction, and the underlying reason for this is unknown [46]. Most patients in our analysis were on tacrolimus or MMF, and CNIs were frequently modified post-infection. No studies reported decreased mortality with a change in immunosuppressants; a study by Colmenero et al. hypothesised that MMF might exacerbate the cytotoxic effects of SARS-CoV-2 on lymphocytes and worsen the immune response [22]. In contrast, Belli et al. reported reduced mortality with tacrolimus use [30]. The probable role of tacrolimus was attributed to immunosuppressive effects on T-cells rather than inhibition of viral replication. Coronavirus depends on active immunophilin pathways for replication. Tacrolimus may inhibit replication by binding immunophilin FK-506-binding proteins [30]. Further, there are reports of improved outcomes with continued immunosuppression during COVID-19 [47,48]. Extensive studies are required to ascertain these findings.

Recently, Thieme et al. found comparable cellular and humoral immune responses to SARS-CoV-2 infection in SOT recipients on immunosuppressants vs non-immunosuppressed (non-transplant) patients [49]. Over-activation of the complement system and a prolonged inflammatory response due to discordant expression of type I and II cytokines results in a cytokine storm and unfavourable outcomes in SARS-CoV-2 infection [50]. Immunosuppressive drugs, which alter the immune status and suppress the cytokine storms, have been tried in non-SOT COVID-19 infected patients with limited success. Furthermore, autoimmune hepatitis (non-LT) patients infected with SARS-CoV-2 are protected from liver injury when immunosuppression is continued [51,52]. However, it is currently recommended to lower the overall immunosuppression (especially anti-metabolites) in LT patients infected with COVID-19, similar to managing infections in transplant recipients to reduce the risk of superinfection [11,53].

The availability of COVID-19 vaccines could boost SOT programs by mitigating the likelihood of severe COVID-19 infection. Data on the safety and efficacy of vaccines in waitlisted candidates and LT recipients are in progress [54].

The major limitation of this meta-analysis is the lack of uniformity in reporting the effects of changes in immunosuppression on mortality. The effect of steroids or triple immunosuppression on the outcomes has not been reported. Studies should focus on the effects of high-dose steroids and modified immunosuppression in infected LT patients. Since some study data was from registries, there may be a risk of re-analysing the same patients across multiple studies, which is challenging to overcome. However, data from registries give a global overview of the disease and cannot be excluded [20]. Some of the reported 95% CIs are wide, suggesting that a lack of statistical significance may not necessarily exclude the possibility of clinical relevance. This may be due to uneven distributions of patients between groups and insufficient power to detect differences. We tried to overcome this limitation with sensitivity analyses.

This study comprehensively describes COVID-19 clinical features, disease course, and outcomes in LT recipients. Prior meta-analyses were limited to case reports and case series, while our review is the largest to date [43,55,56]. We demonstrated that the available evidence points to the cautious reestablishment of transplant programs globally. With appropriate immunosuppression and equivalent outcomes, COVID-19 can be managed suitably in LT recipients.

## Data sharing statement

Data used for meta-analysis will be made available on request to the corresponding author.

## Contributors

AVK and HVT conceptualised and designed the study, and retrieved data; MP and PK rechecked the data; AVK and RC conducted study analyses; AVK, MP, and HVT did the initial drafting; PNR and DNR supervised the study; JPA, MS, RC, KK, PNR, and DNR critically assessed the data and provided intellectual inputs. All members approved the final draft.

## Funding

None.

## Declaration of Competing Interest

We declare no competing interests.

## Appendix Supplementary materials

Image, application 1

Image, application 2

Image, application 3
