# Supplementary material for: Impact of COVID-19 on liver transplant recipients–A systematic review and meta-analysis
Source: eClinicalMedicine. 2021 Jul 13;38:101025. doi: 10.1016/j.eclinm.2021.101025 (PMC8276632; doi:10.1016/j.eclinm.2021.101025)
Supplement: Supplementary file 1 [file mmc1.docx]

**Supplementary material for the article titled “****Systematic Review with Meta-Analysis: Impact of COVID-19 on Liver Transplant Recipients.”**

**Supplementary Table 1: Characteristics of non-liver transplant patients (Controls) infected with COVID-19**

| **Serial no** | **First author, Country, Centres. ^Reference no.^** | n | Age | Males (n, %) | Comorbidities (n, %) | No. of patients in immunosuppression |
| --- | --- | --- | --- | --- | --- | --- |
| 1 | Webb et al., UK, Multicentre. ^24^ | 627 | 73 (55–84) | 329 (52%) | Hypertension-241 (38%)  Diabetes-144 (23%)  Cardiovascular disease-275 (43·85%)  Pulmonary disease- 160 (25·6%)  CKD-95 (15·2%)  Obesity (BMI >30 kg/m^2^)-158 (25%)  Cirrhosis-6 (1%)  Other organ transplant-3 (0·5%) | Steroids-17 (2·7%)  Calcineurin inhibitors-6 (0·9%)  Anti-metabolites-7 (1·1%) |
| 2 | Rabiee et al.,  USA, Multicentre. ^29^ | 375 chronic liver disease patients | 60 (IQR,19) | 57% | Hypertension- 222 (59·3%)  Diabetes- 177 (47·1%)  Obesity-140 (37·5%) | - |
| 3 | Mansoor et al., USA, Multicentre. ^16^ | 125 | 59·83 ±14·71 | 85 (68%) | Hypertension-23 (18%)  CKD-26 (21%)  Diabetes-20 (16%)  Pulmonary disease-10 (8%)  Cardiovascular-30 (24%) | Steroids-20 (16%)  Calcineurin inhibitors- 10 (8%)  Anti-metabolites (azathioprine)-10 (8%) |
| 4 | Trapani et al., Italy, Multicentre.^31^ | 238,876 | 61·4 ± 21·11 | 109,261 (45·7%) | - | - |
| 5 | Polak et al. Netherlands, Multicentre. ^32^ | 57 LT candidates. | 59 ± 15 | 93% | - | - |

CKD-chronic kidney disease; BMI-body mass index

**Supplementary Table 2: Comparison of comorbidities among studies reporting mortality among liver transplant (LT) recipients and non-LT patients.**

| Comorbidities | a. All included studies | | | b. Studies comparing mortality | | |
| --- | --- | --- | --- | --- | --- | --- |
|  | LT recipients | Non-LT patients | *P*-value | LT recipients | Non-LT patients | *P*-value |
| Hypertension | 44·3% (485/1,095) | 43% (486/1,127) | 0·58 | 33·34% (92/276) | 35·1% (264/752) | 0·6 |
| Diabetes | 39·4% (431/1,095) | 30·2% (341/1,127) | <0·001 | 30·8% (85/276) | 21·8% (164/752) | 0·003 |
| Kidney disease | 13·7% (150/1,095) | 16% (121/752) | 0·15 | 20% (25/125) | 16% (121/752) | 0·27 |
| Obesity | 23·6% (158/670) | 33·43% (335/1, 002) | <0·001 | 29% (44/151) | 25% (158/627) | 0·32 |
| Cardiovascular disease | 16·43% (180/1,095) | 40·55% (305/752) | <0·001 | 15·2% (42/276) | 40·55% (305/752) | <0·001 |
| Pulmonary disease | 9% (99/1,095) | 22·6% (170/752) | <0·001 | 6·5% (18/276) | 22·6% (170/752) | <0·001 |
| ACEi | 27% (70/259) | - |  |  |  |  |

ACEi-angiotensin-converting enzyme inhibitors.

**Supplementary Table 3: Clinical features, outcomes, and treatment received for COVID-19 in LT recipients**

| **Serial no.** | **First author, Country, Centres. ^Reference no.^** | **Time to infection after LT (median)** | **Symptoms** | **Markers** | **Outcome** | **Treatment of COVID-19** | **Cause of death** |
| --- | --- | --- | --- | --- | --- | --- | --- |
| 1 | Colmenero et al., Spain, Multicentre. ^22^ | 105 months (IQR-35–168). | Fever-83 (74·8%);  Dyspnoea-46 (41·4%)  Cough-78 (70·3%);  GI symptoms- 38 (34·2%)  Asymptomatic-7 (6·3%)¶ | Ferritin-1050 (517–3299) ng/ml  D-dimer-600 (345–1630) ng/ml  Lymphocyte count-670 (430–1040) cells/µL | Mortality-20/111 (18%)  Respiratory support-22/111 (19·8%)  Graft dysfunction-3/111 (2·7%);  Admitted- 96/111 (86·5%)  Transaminases- 16/111 (14·7%);  ICU-12 (10·8%)  Severe disease-31·5% (35/111) | Azithromycin-60/96 (62·5%);  HCQ-88/96 (91·7%);  LPV/R-40/96 (41·7%)  IFN-β -3/96 (3·1%)  Remdesivir-1/96 (1%)  Tocilizumab-15/96 (15·6%)  Steroids-12/96 (12·5%) | COVID-19 related |
| 2 | Becchetti et al., Switzerland, Multicentre. ^17^ | 6 years (2–13) | Fever-44 (79%);  Cough-31 (55%)  Dyspnoea-26 (46%);  Fatigue/malaise-32 (56%);  GI-18 (33%)  Anosmia-4 (7%) | Ferritin-567 (171–1194) ng/ml (n=13)  IL-6-93 (59–288) pg/L (n=9)  D-dimer-871 (380–1739) ng/ml (n=17)  TLC-4400 (3330–6000) cells//µL (n=51)  Lymphocyte-790 (400–1100) cells//µL(n=49) | Mortality-7/57 (12%)  ICU-4 (7%)  Ventilation-12 (21%)  Hospital stay- 41 (72%)  Severe disease-19·3% (11/57)  Bacterial infection-9/57 (16%) | Azithro-15/57 (27%)  >1 antibitoic-16/57 (29%)  Remdesivir-1/57 (2%)  LPV/r-3/57 (5%)  Darunavir/cobicistat-1/57 (2%)  Tocilizumab-1 (2%)  Rituximab-2 (2%)  Ruxolitinib-1 (2%)  HCQ- 24/57 (44%)  Oxygen therapy-18/57 (32%) | ARDS.  (5 out 7 had a history of cancer) |
| 3 | Lee et al., USA, Single centre. ^23^ | 3·8 (0·02–28·2) years | Fever- 23/38 (61%);  Cough-21/38 (55%);  Dyspnea- 13/38 (34%);  Myalgias-9/38 (24%)  Malaise-11/38 (29%);  Rhinorrohea-3/38 (8%);  GI-16/38 (42%);  Ansomia-1/38 (3%);  Asymptomatic-2/38 (5%)ǂ | Ferritin-986 (36–4677) ng/mL  IL-6-66·3 (12·5–218) pg/mL  CRP-66 (6·2–430·3) mg/L  D-dimer-1670 (270–8620) ng/ml  Lymphocytes-600 (200–5600) (n=24) | Mortality -7/38 (18%)  Ventilation-8/38(21%)  Hospitalized- 27/38 (72%)  ICU-8/38 (21%)  Elevated liver enzymes-7·9% (3/38)  Severe disease-11/24 (46%)  AKI-13/24 (54%)  Ischemic stroke-1/38 (2·6%)  Bacterial infections-2/38 (5·26%)  Fungal infection-1 | Oxygen-18/24 (75%)  HCQ ±Azithromycin-18/24 (75%)  Steroids-5/24 (21%)  Anticoagulation-8/24 (33%) | All patients had diffuse lung infiltrates (Pulmonary failure)  2 patients developed bacteremia (one had concomitant fungemia, and one developed ischemic stroke) |
| 4 | Webb et al., UK, Multicentre. ^24^ | 5 years (2–11) | GI- 45/149 (30·2%)  Respiratory- 114/149 (77%) |  | Mortality-28/151 (18·5%) vs 167/627 (27%)  Ventilation-30/151 (20%) vs 32/627 (5%)  Hospitalized-124/151 (82%) vs 474/627 (76%)  ICU-43/151 (28%) vs 52/627 (8%)  Duration of hospital stay-11 (6-16) (n=79) vs 8 (3-13 (n=316)  Elevated liver chemistries – total-65/121 (53·71%) vs 206/448 (46%)  Severe-20%  AKI stage 2 -27/129 (21%) in Tx cohort vs 94/537 (18%) in non-LT group.  Stage 3-22/130 (17%) vs 74/537 (14%) | 49 (32%) received specific therapy.  Azithromycin-1 (1%)  HCQ-38/151 (25%)  LPV/r-9 (6%)  Remdesivir- 6 (4%)  Oseltamivir-3 (2%)  Sofosbuvir -1 (1%)  Anakinra- 2 (1%)  Plasma-2 (1%)  Tocilizumab-2 (1%) Heparin- 1 (1%) | Transplant Cohort  Liver related-0  Respiratory-21(75%)  Cardiogenic-2(7%)  Others-5(18%)  Non-transplant-  Respiratory-146(89%)  Cardiac-3 (2%)  Other-15 (9%) |
| 5 | Malekhosseini. et al  Iran, Single centre. ^25^ | 35·4 months (13·7–70·6) | Fever-28 (42·4)  Myalgia-30 (45·5  Cough-27 (40·9  Fatigue-27 (40·9)  Dyspnoea-20 (30·3)  Anorexia-21 (31·8)  Headache-20 (30·3)  Nausea/vomiting-13 (19·7)  Sputum-6 (9·1)  Rhinorrhoea-8 (12·1)  Abdominal pain-7 (10·6)  Sore throat-12 (18·2)  Diarhoea-13 (19·7) | CRP-43·9 ± 55 mg/L  TLC-4876±2892 cells//µL | Mortality-12/66 (18·2)  Ventilation (IV+NIV)-10/66 (15·2%)  ICU admission rate-12/66 (19%)  Hospital stay-8·9± 6·3  Elevated liver enzymes – 12/66 (19%)  Severe disease -17 (33·3%)  Increased creatinine-14/66 (42·4%) | Azithromycin-20/66 (30%), Cotrimoxazole-3(4·5%)  Vancomycin-2 (3%)  Imipenem-3 (4·5%)  HCQ-22 (33·4%)  Lpv/r-1 (1·5%)  Oseltamivir- 2 (3%)  Fluconazole-2 (3%)  Guanfacine-4 (6%) | COVID-19 related death |
| 6 | Patrono et al., Italy,  Single centre. ^26^ | <1 year-3  >1 year-7 | Fever-7(70%)  Diarrhoea (GI)-3/10(30%)  Cough-3/10(30%)  Myalgia—1/10 (10%)  Anorexia-1/10 (10%)  Sore throat-1 |  | Mortality-2/10 (20%)  NIV-2/10 (20%)  Hospitalization-9/10(90%)  Severe disease-2/10 (20%) | HCQ-6/10 (60%)  Steroids-3/10 (30%)  Oxygen-6/10 (60%)  Lpv/r-1/10 (10%)  Darunavir/r-1 (10%) | COVID-19 related-10%  Unrelated cause-10% |
| 7 | Loinaz et al., Italy,  Single centre. ^27^ | 83 (20–183) months. | Fever-8/19 (44%)  Cough-16/19(84·2%)  Chest pain-6/19-(31·5%)  Dyspnoea-9 (47·4%)  GI-9/19 (6 diarrhoea and 3 nausea vomiting)  Anosmia or dysgeusia-2/19 |  | Mortality-2/19-( 10·5%)  NIV+IV-2/19 (10·5%)  ICU-2/19  Hospitalization-14/19(73·68%)  Thrombotic complications- 2/19 (10·5%)  Elevated liver enzymes-6/19 (31·6%) (4 mild to moderate and 2 severe)  Severe-3 (16%) | 12 received therapy, and 7 did not receive any.  HCQ-9/19  LPV/r-2  IFN-β -1  Tocilizumab-2  Steroids-2  Oxygen-7/19 | Respiratory failure |
| 8 | Dhampalwar et al.,  India, Single centre.^28^ | 3 within 3 months of  LT. 9 developed after 18months. | fever (83·3%),  cough (41·7%), and sore throat (41·7%)  Asymptomatic-1/12¶¶ |  | Mortality/graft dysfunction/ ICU and IV/Severe disease- 8·3% (1/12)  AKI-16·7% (2/12) |  | Multiorgan failure |
| 9 | Rabiee et al.,  USA, Multicentre. ^29^ | 4 (IQR-11) years | - |  | Mortality(overall) -25/112 (22·3%)  MV-26/112 (23·2%)  Hospitalized-81/112 (72·3%)  ICU-30/112 (26·8%)  Liver injury-34·6% (112) vs 47·5% among 375 CLD patients.  Acute rejection-1  Severe disease-22·3% (26/112) | Azithromycin-31 (27·7%)  Remdesivir- 3 (2·7%)  Steroids- 4 (3·6%)  HCQ-42 (37·5%)  HCQ+Azitho-26 (23·2%)  Oygen-59/112 (52·7%) | COVID-19 related |
| 10 | Mansoor et al., USA, Multicentre. ^16^ |  | Fever-12 (10%)  Cough-10 (8%)  GI-20 (16%)  (Diarrhea-10 and Nausea vomiting-10)  Abdominal pain-10 (8%)  Dyspnea-10 (8%)  Fatigue-10 (8%)  Sore throat-10 (8%) | Ferritin-9333·93 ± 35,380·02 (LT) vs 23,411·78 3 ± 89,271·63 ng/ml (non-LT)  IL-6-31·6 ± 0 vs 224·37 ± 277·5 pg/ml (non-LT)  CRP-48·17 ± 64·48 vs 102·94 ± 92·72 mg/dl  D-dimer-2·81 ± 5·97 vs 3·31 ± 2·22 FEU  TLC-LT-6560 ±5420 vs 6760 ± 2700 cells//µL  Lymphocyte-790±280 vs 410 ±270 cells//µL | Mortality-10/126 (8%) vs 10/125 (8%)  Hospitalization-50 (40%) vs 29/125 (23%) in non-LT  ICU-10 (8%) vs 11 (9%)  Thrombosis-10 (8%) vs 10/125 (8%) in non-LT  Severe disease-8% |  | Mortality after COVID-19 |
| 11 | Belli et al., Europe, Multicentre.^30^ | 8 (3·1–15) years | Fever-190 (78·2%)  Cough- 143 (58·8%)  Dyspnea-82 (33·7%)  Muscle pain or asthenia in 90 (37·0%)  Anosmia or dysgeusia- 21 (8·6%)  GI (diarrhea)-55 (22·6%)  Confusion-15 (6·17%) | TLC-5200 (4000–6700) cells//µL | Overall mortality- 49/243 (20·2%)  Ventilation- 51/243 (21%)  Total hospitalization -204/243 (84%)  ICU- 39/243 (16%)  Hospitalization-167/243 (69%)  Thrombotic events-7/204 (3·4%)  Liver injury-56 (23%)  Acute rejection-3  Severe disease- 51/243 (21%)  Renal replacement therapy-21/243 (8·64%)  Bacterial infection-18/243 (7·41%)  Fungal-6 /243(2·57%) | None-94/243 (38·68%)  Azythromycin-67 (27·57)  Lpv/r-41/243 (16·87)  Remdesevir-1 (0·41)  HCQ- 116/243 (47·74)  High-dose steroids-34 (13·99)  Tocilizumab-15 (6·17)  Other-24 (9·88  Oxygen-86 (62·77%)  LMWH-117 | Refractory pneumonia-38 (77·5%)  Liver related with lung failure-1 (2·04%) and without lung failure- 3 (6·12%)  Others-7 (15%) |
| 12 | Trapani et al., Italy, Multicentre.^31^ | Median-6·1 years |  |  | Mortality-14/89 (15·7%) vs 33,972/238895 (14·2%)  Hospital-48/89 (53·9%) vs77069/238895 (32·3%)  ICU-5/89 (5·6%) vs 17506/238895 (7·3%)  Severe-53/89 (59·6%) in LT vs 94575/238895 (39·6%) in non -SOT· |  | Death within 60 days after COVID-19. |
| 13 | Polak et al. Netherlands, Multicentre. ^32^ |  |  |  | Mortality-36/244 (15%) vs/ 10/57 (18%) in LT candidates  ICU-33/244 (13·52%) vs 8/57 (14%) (in LT candidates)  Severe disease-36 |  | Mortality due to COVID-19 or its complications |
| 14 | Gruttadauria et al., Italy, Multicentre. ^33^ | <1 year -5 |  |  | Mortality-5/24 (21%)  ICU-3/24 (12·5%)  Hospitalization- 17 (71%)  Severe disease-21% |  | Mortality due to COVID-19 |
| 15 | Pereira et al., USA, Multicentre.^34^ |  |  |  | Severe disease-4/13 (31%)  Thrombotic events- 0/13 |  | Mortality due to COVID-19 related complications (n not mentioned) |
| 16 | Ali et al., Saudi Arabia, Single centre. ^35^ | 5·1 (0·1–14·5) years | Asymptomatic-4 ǂǂ | Ferritin-322 (121–1486) ng/ml (n=15)  CRP-117 (6–168) mg/L  D-dimer-1230 (420–5800) ng/ml | Mortality-0/15  ICU-4 (26·67%)  Admitted-10 (66·7%)  NIV+IV-0  AKI-3/15 (20%)  Severe disease-2/15 (13·34%) | HCQ-8  Azithromycin-8  Tocilizumab-3  Dexamethosone-3 | No deaths |
| 17 | Kates et al., USA, Multicentre. ^36^ | 5 (2–11) years | Fever-34/73 (46·6%)  Cough-50 (68·5%)  Dyspnea-40 (54·8%)  GI-29 (39·7%)  URI-24 (32·9%)  Fatigue-32 (43·8%)  Myalgia-21 (28·8%) |  | Mortality-15/73 (20·6%)  Hospitalization-47 (64·4%)  ICU-23 (32·9%)  IV-20 (27·4%)  AKI-24 (32·9%)  Elevated liver chemistries (>3 ULN)-6 (8·2%)  Thrombotic-2 (2·7%)  Acute cellular rejection-2 (2·7%)  Antibody rejection-1 (1·4%)  Severe disease- 20 (27·4%) |  | 28-day mortality after COVID-19 diagnosis |
| 18 | Dumortier et al. France, Multicentre ^37^ | 84·9 (34–168) months | Fever-55 (60·4%)  Cough-51 (56%)  GI-25 (27·57%)  (Diarrhea-19 and vomiting-6)  Dyspnea-45 (49·5%)  Rhinitis-13 (14·3%)  Anosmia-9 (9·9%)  Neurologic (includes headache)-29 (31·86%)  Myalgia- 28 (30·8%) | CRP-70 (28–122) mg/L (n-46)  Lymphocyte- 700 (550–1080) cells//µL (n=38) | Mortality-19 /91 (20%)  Severe disease-30/91 (33%)  Thromboembolic event-9 (13·4%)  AKI-32/67 (47·8%)  Mechanical ventilation-17/67 (25·4%)  RRT-11 (16·4%)  Hospitalization- 67/91 (73·62%)  Bacterial infection-17/67  Fungal-1/67  Both bacterial and fungal-1/67  Elevated liver chemistries-11/67 (16·4%)  Graft dysfunction-0 | Azithromycin-16/67 (23·9%)  Remdesivir- 2 (3%)  Steroids- 6 (9%)  HCQ-14 (20·9%)  LPV/r-2 (3%)  Other antibtioics- 40 (59·7%)  Tocilizumab-1 (1·5%)  Oxygen-59·7% | ?COVID-19 related |

GI-gastrointestinal; ICU-intensive care unit; LPV/r-lopinavir with ritonavir; IFN-β-interferon- beta; HCQ-hydroxychloroquine; IV-invasive ventilation; NIV-non-invasive ventilation; CLD-chronic liver disease; LT-liver transplant; LMWH-low molecular weight heparin; AKI-acute kidney injury; URI-upper respiratory tract infection; IL-interleukin, TLC-total leucocyte count, CRP-C-reactive protein, ULN-upper limit of normal.

¶ Asymptomatic patients were tested for COVID-19 because of incidental radiological findings (n = 2) or after a high-risk contact (n = 5).

ǂ One individual was detected prior to endoscopy and one prior to hospital admission (testing done as a part of hospital protocol).

¶¶ One individual was detected positive when investigated because of contact exposure.

ǂǂ Asymptomatic patients were tested if they had a history of contact with a confirmed case of COVID-19, before undergoing a procedure or before admission to the hospital for any reason.

**Supplementary Table 4: Immunosuppression before and after COVID-19 infection.**

| **Serial no** | **First author, Country, Centres. ^Reference no.^** | **Immunosuppression before infection** | **Change in immunosuppression after infection** |
| --- | --- | --- | --- |
| 1 | Colmenero et al., Spain, Multicentre.^22^ | CNI-34 (30·6%)  CNI+MMF-29 (26·1%)  CNI+Everolimus-37 (33·3%)  None-2 (1·8%)  Tac-66 (59·5%)  CsA-6 (5·4%)  MMF- 57 (51·4%)  Everolimus-23 (20·7%)  Steroid-24 (21·8%) |  |
| 2 | Becchetti et al., Switzerland, Multicentre.^17^ | CNIs-16 (28%)  CsA-3 (5%)  Tac-13 (29%)  mTORi-2 (4%)  MMF-2 (3%)  CNIs+MMF-21 (37%)  CNI+mTORi-3 (5%)  CNI+steroids-9 (16%)  CNIs+AZA-1 (2%)  mTORi+MMF-2 (3%) | No change-25 (43·85%)  Reduction in CNIs-12 (21%) and MMF-1 (1·7%%)  Discontinued CNIs in 7 (12·2%), mTOR in 3 (5·2%), and MMF in 8 (14%) |
| 3 | Lee et al., USA, Single centre.^23^ | Tac-37/38 (97%)  CsA-1/38 (3%)  Everolimus-1/38 (3%)  MMF-19/38 (50%)  Steroids- 15/38 (39%) | Decrease -19/24 (79%)  CNI reduction-15/23 (65%)  MMF reduction- 13/13 (100%)  Steroid- 2/12 (17%) |
| 4 | Webb et al., UK, Multicentre. ^24^ | CNI-135/150 (89%)  Tac- 127/150 (84%)  CsA- 8 (5%)  Antimetaolite-90/150 (60%)  (MMF-77/150 (51%) Aza- 13/150 (9%)  Sirolimus- 7/150 (5%)  Steroids-67/150 (44%) |  |
| 5 | Malekhosseini. et al  Iran, Single centre. ^25^ | Prednisolone- 39 (59·1%)  MMF-55 (83·4%)  TAC- 53 (80·3%)  Sirolimus- 5 (7·6%)  CsA- 4 (6·1%)  Everolimus-6 (9%) | Not mentioned |
| 6 | Patrono et al., Italy,  Single centre. ^26^ | Tac-10/10(100%)  MMF-6/10(60%)  Everolimus-2/10(20%)  Prednisolone-3/10(30%) | Modified in 70% (7/10)  Stopped Tac-4/10 (40%)  ReducedTac-1/10 (10%)  Stop MMF-2/6 (34%)  Decrease MMF-1/6 (17%) |
| 7 | Loinaz et al., Italy,  Single centre. ^27^ | Tac-8/19 (42%),  mTORi-4  MMF-8  Aza-1  Everolimus-2 | 3/19 (16%) change in IS  Everolimus stopped and changed to low dose long-acting Tac.  MMF was stopped in 1 |
| 8 | Dhampalwar et al.,  India, Single centre. ^28^ | Tac- 10 (83·3%)  Everolimus-1 (8·3%)  CsA-1 (8,3%) | The dosage of MMF was reduced in most patients with a diagnosis of COVID-19 |
| 9 | Rabiee et al.,  USA, Multicentre. ^29^ | Tac-103/112 (91·9%)  CsA-7/112 (6·3%)  MMF-56/112 (50%)  Aza-1 (0·9%)  Steroids-34/112 (30·4%)  mTORi-4 (3·6%)  Other-3 (2·7%) | Modified- 52/112 (47%)  Stopped MMF-27/56 (48·21%)  Reduction in Tac-21/103 (20·3%)  Stop Tac-4/103 (4%) |
| 10 | Mansoor et al., USA, Multicentre. ^16^ | Tac-77 (61%)  CsA-10 (8%)  Prednisolone-49 (39%)  Hydrocort-11 (9%) MMF-47 (37%)  Aza-10 (8%)  mTORi-10 (16%)  (Sirolimus-10 (8%)  Everolimus-10 (8%)  Basiliximab-10 (8%) |  |
| 11 | Belli et al., Europe, Multicentre.^30^ | TAC - 162 (66·67%)  MMF-119 (48·97%)  Steroids-56 (23·05%)  mTOR-37 (15·23%)  CsA-29 (11·93%)  Other-1 (0·41%) | Immunosuppression changes-97/243 (39·92%)  Stopped CNI-16 (6·58%)  25%-50% reduction in CNI-38 (15·64%)  Stopped antimetabolites-35 (14·40%)  Stopped mTOR inhibitors-10 (4·12%)  Other-6 (2·47%) |
| 12 | Polak et al. Netherlands, Multicentre. ^32^ |  | 86% modified IS (details not mentioned) |
| 13 | Ali et al., Saudi Arabia, Single centre. ^35^ | Steroids-5 (33·3%)  Tac-14 (93·3%)  MMF/Aza-9 (60%) |  |
| 14 | Kates et al., USA, Multicentre. ^36^ | CNI, antimetabolite and steroids-23 (31·5%)  CNI and steroids-6 (8·2%)  CNI and antimetabolite-15 (20·6%)  mTORi-3 (4·1%)  Other-30 (41·1%) |  |
| 18 | Dumortier et al. France, Multicentre.^37^ | Tac-70/90 (77·8%)  CsA-6/90 (6·7%)  MMF-53/91 (58·2%)  Azathioprin-3/91 (3·3%)  mTORi- 14/91 (15·4%)  Steroids-16/91 (17·6%)  Belatacept-2 /91(2·2%) | Immunosuppresion modidfed in 30/67 (44·7%)  CNI withdrawal-12·5% (7/56)  Antimetabolite withdewal-41·9% (18/43)  mTORi withdrawal- 30% (3/10)  belatacept withdrawal-100% (2/2) |

CNI-calcineurin inhibitors; MMF-mycophenolate mofetil; Tac-tacrolimus; CsA-cyclosporine; mTORi-mechanistic target of rapamycin inhibitor; Aza-azathioprine

**Supplementary Table 5: New-castle Ottawa scale (NOS) score for each included study.**

| **Serial no** | **First author, Country, Centres. ^Reference no.^** | **Type of study, cohort studies**  **(retrospective and prospective)** | **Selection** | **Comparability** | **Exposure** | **Score** |
| --- | --- | --- | --- | --- | --- | --- |
| 1 | Colmenero et al., Spain, Multicentre. ^22^ | Prospective cohort study | ☆☆☆ | ☆ | ☆☆☆ | 7 |
| 2 | Becchetti et al., Switzerland, Multicentre. ^17^ | Prospective cohort study | ☆☆☆ | ☆ | ☆☆☆ | 8 |
| 3 | Lee et al., USA, Single centre. ^23^ | Retrospective cohort study | ☆☆ | ☆ | ☆☆☆ | 6 |
| 4 | Webb et al., UK, Multicentre. ^24^ | Prospective cohort study | ☆☆☆☆ | ☆ ☆ | ☆☆☆ | 9 |
| 5 | Malekhosseini. et al  Iran, Single centre. ^25^ | Prospective cohort study | ☆☆☆ | ☆ | ☆☆☆ | 7 |
| 6 | Patrono et al., Italy,  Single centre. ^26^ | Case series | ☆☆ | ☆ | ☆☆ | 5 |
| 7 | Loinaz et al., Italy,  Single centre. ^27^ | Prospective cohort study | ☆☆☆ | ☆ | ☆☆ | 7 |
| 8 | Dhampalwar et al.,  India, Single centre.^28^ | Case series | ☆☆ | ☆ | ☆☆ | 5 |
| 9 | Rabiee et al.,  USA, Multicentre. ^29^ | Prospective cohort study | ☆☆☆☆ | ☆☆ | ☆☆☆ | 9 |
| 10 | Mansoor et al., USA, Multicentre. ^16^ | Retrospective cohort study | ☆☆☆☆ | ☆☆ | ☆☆☆ | 9 |
| 11 | Belli et al., Europe, Multicentre. ^30^ | Prospective study | ☆☆☆ | ☆ | ☆☆☆ | 7 |
| 12 | Trapani et al., Italy, Multicentre.^31^ | Retrospective study | ☆☆☆ | ☆☆ | ☆☆ | 7 |
| 13 | Polak et al. Netherlands, Multicentre. ^32^ | Prospective study | ☆☆☆ | ☆☆ | ☆☆ | 7 |
| 14 | Gruttadauria et al., Italy, Multicentre. ^33^ | Retrospective study | ☆☆ | ☆ | ☆ ☆ | 5 |
| 15 | Pereira et al., USA, Multicentre.^34^ | Retrospective cohort study | ☆☆ | ☆ | ☆☆☆ | 6 |
| 16 | Ali et al., Saudi Arabia, Single centre. ^35^ | Prospective study | ☆☆☆☆ | ☆ | ☆☆☆ | 8 |
| 17 | Kates et al., USA, Multicentre. ^36^ | Prospective cohort study | ☆☆ | ☆ | ☆☆☆ | 6 |
| 18 | Dumortier et al. France, Multicentre.^37^ | Retrospective cohort study | ☆☆ | ☆ | ☆☆☆ | 6 |

The studies by Colmenero et al., Becchetti et al., Malekhosseini et al., Loinaz et al., and Kates et al. were prospective cohort studies. Belli et al. reported the European Liver and Intestine Transplantation Association (ELITA)/European Liver Transplant Registry (ELTR) based data. Lee et al. reported a retrospective cohort study. Webb et al. reported secure cirrhosis and COVID-Hep registry data, and Rabiee et al. reported data from the prospectively collected COLD registry. Dumortier et al. reported data from the French solid organ transplant registry. Studies by Patrona et al. and Dhampalwar et al. were case series. The studies by Mansoor E, Trapani S, Pereira MR, and Ali T were retrospective cohort based on electronic medical records data. Studies by Polak WG and Gruttadauria S were survey-based data collection from ELTR and the Italian society of organ transplants (SITO).

**Supplementary figure 1: Sensitivity analysis for mortality in COVID-19 infected liver transplant recipients.**


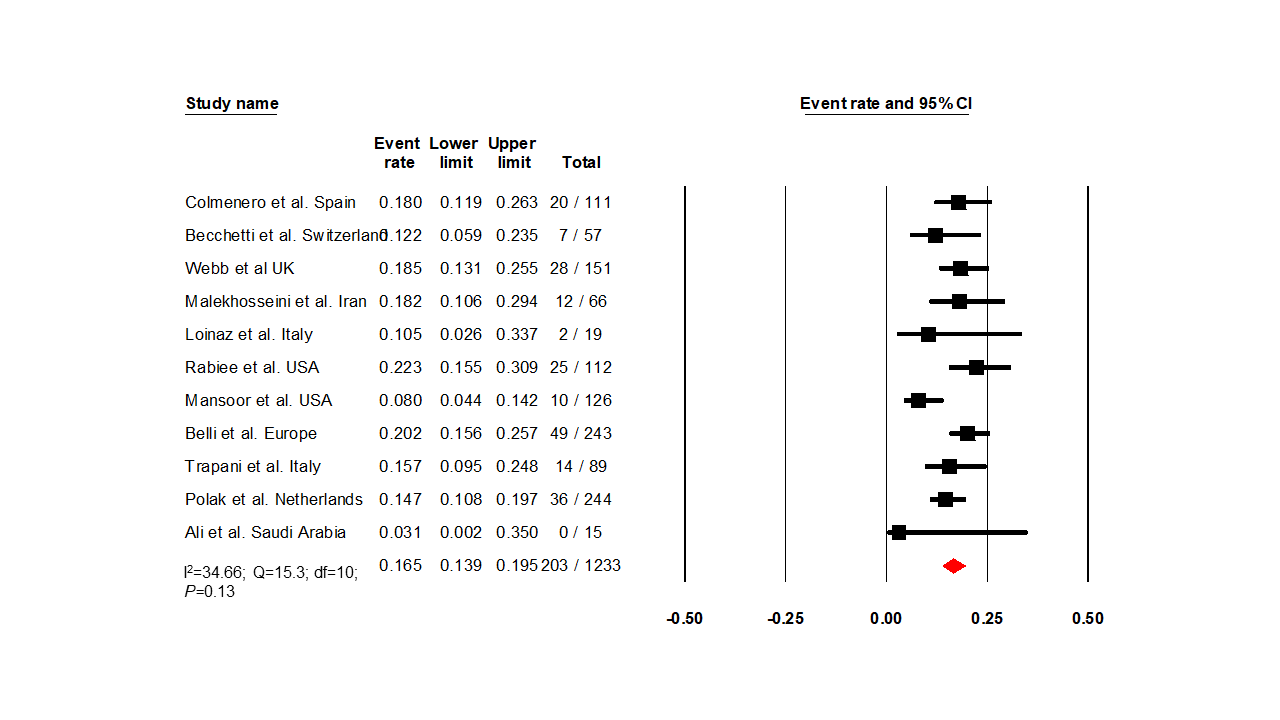


**Supplementary figure 2: Sensitivity analysis of the effect of timing of COVID-19 infection acquisition on outcomes**


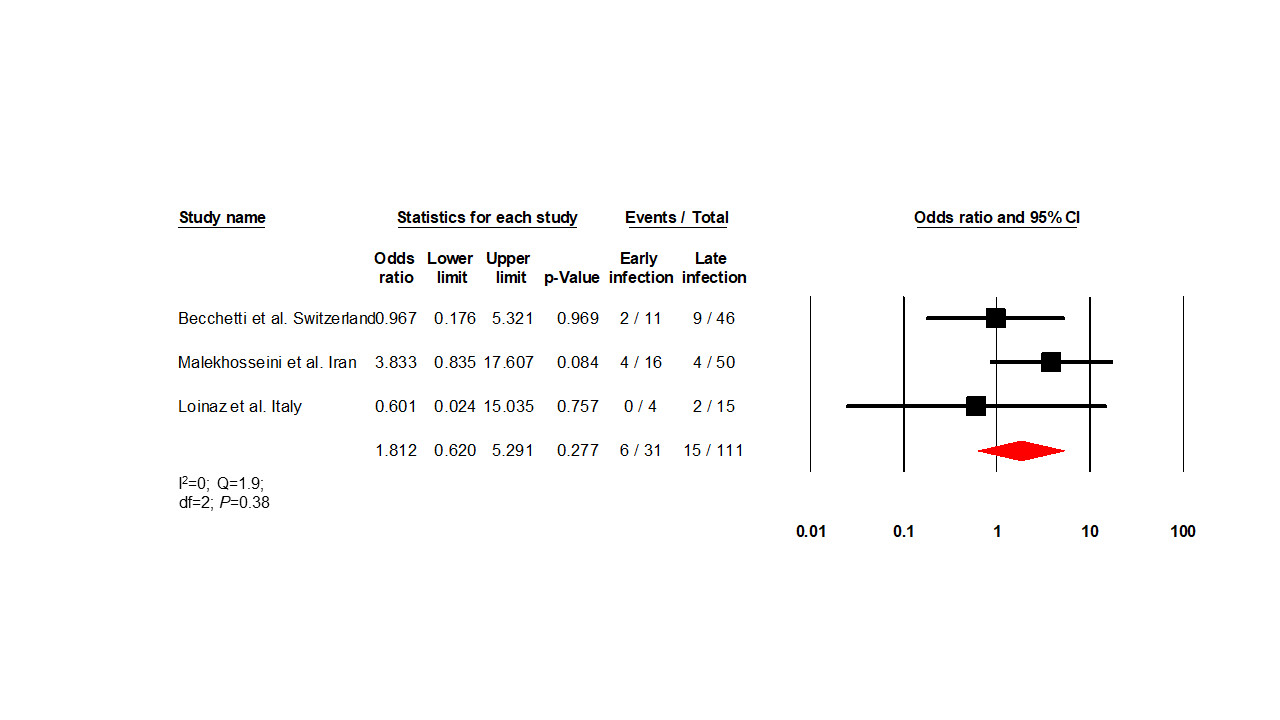


**Supplementary figure 3: Cumulative incidence of hospitalization among COVID-19 infected liver transplant recipients.**


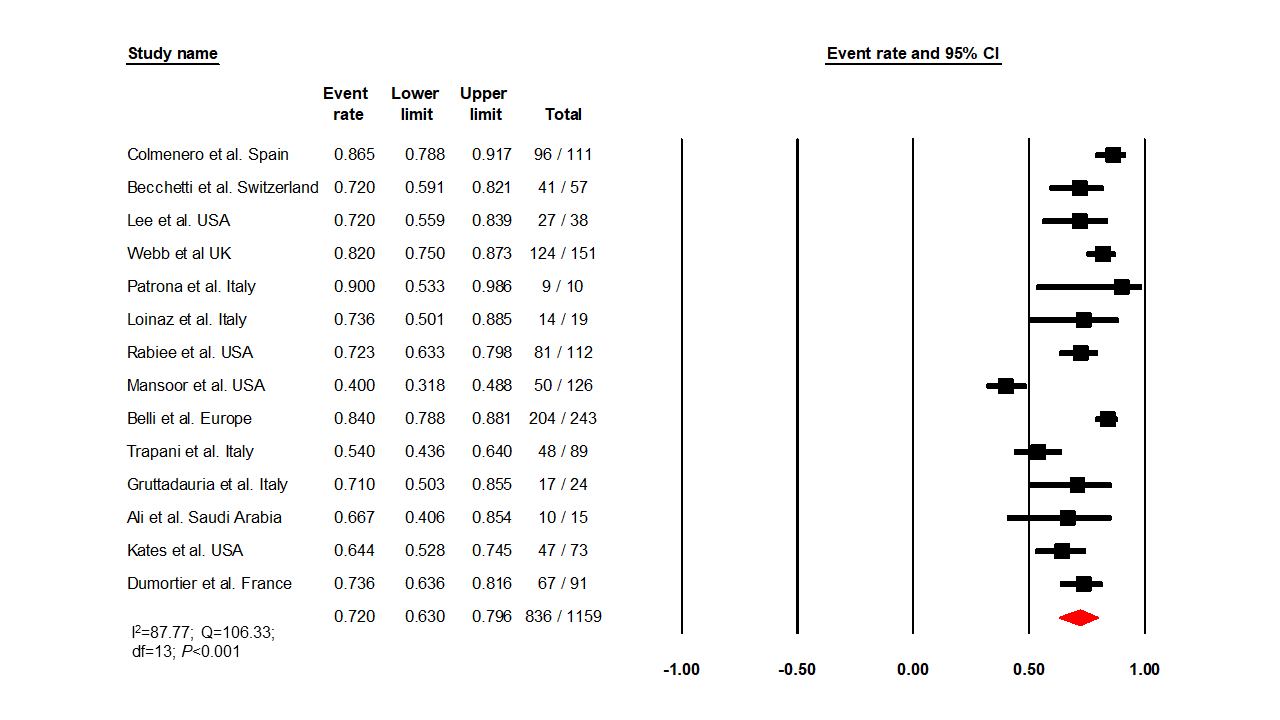


**Supplementary figure 4: Cumulative incidence of intensive care unit admission among COVID-19 infected liver transplant recipients.**


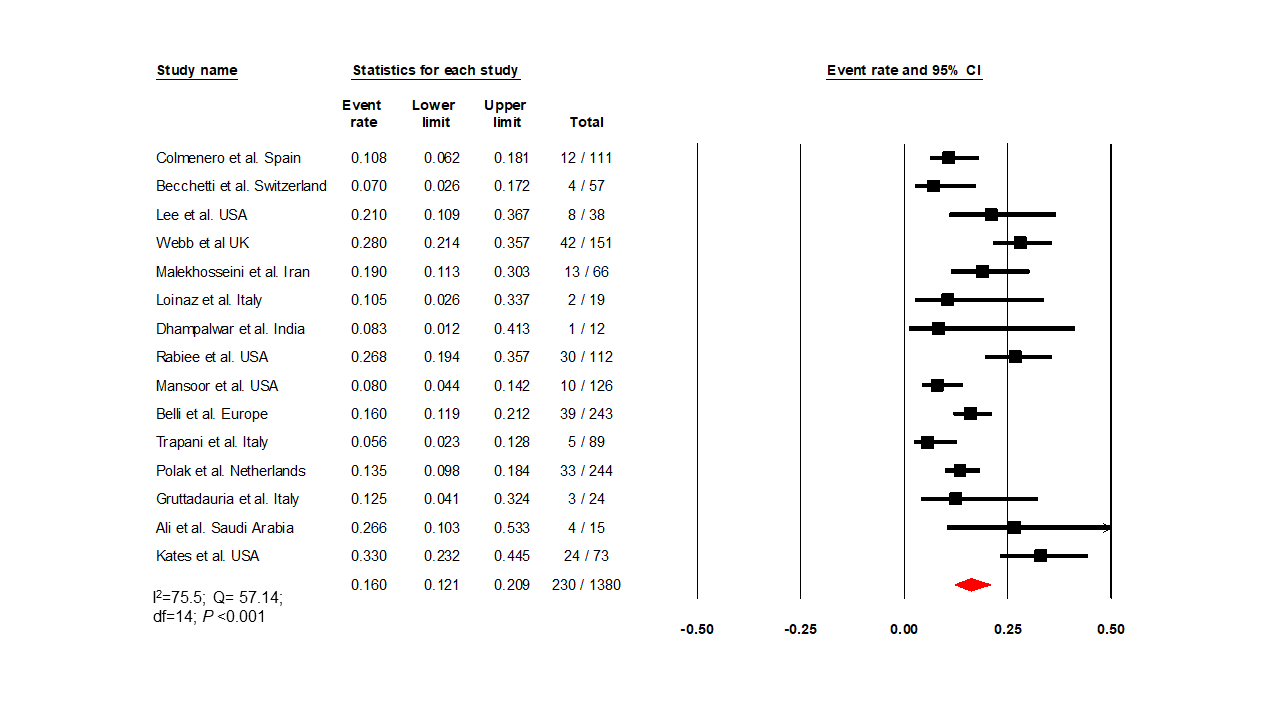


**Supplementary figure 5: Cumulative incidence of COVID-19 infected liver transplant recipients requiring ventilatory support.**


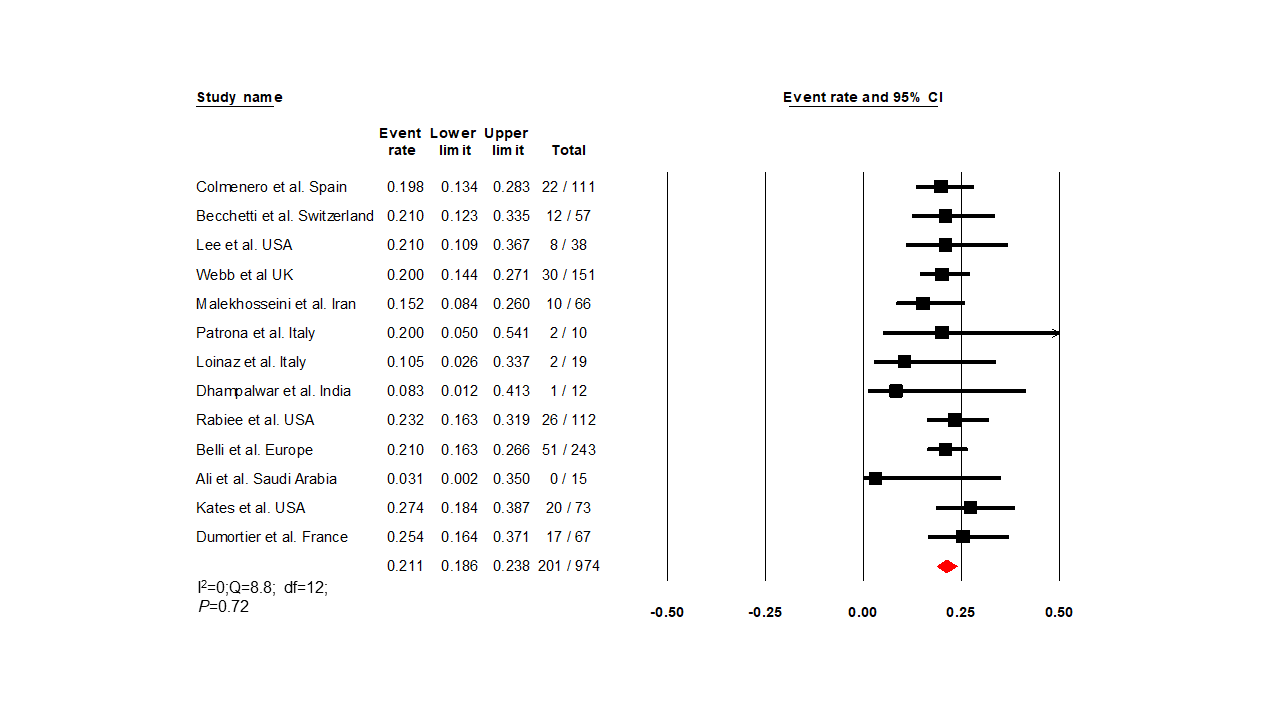


**Supplementary figure 6: Forest plot comparing the proportion of patients with elevated liver chemistries among liver transplant recipients and non-liver transplant recipients.**


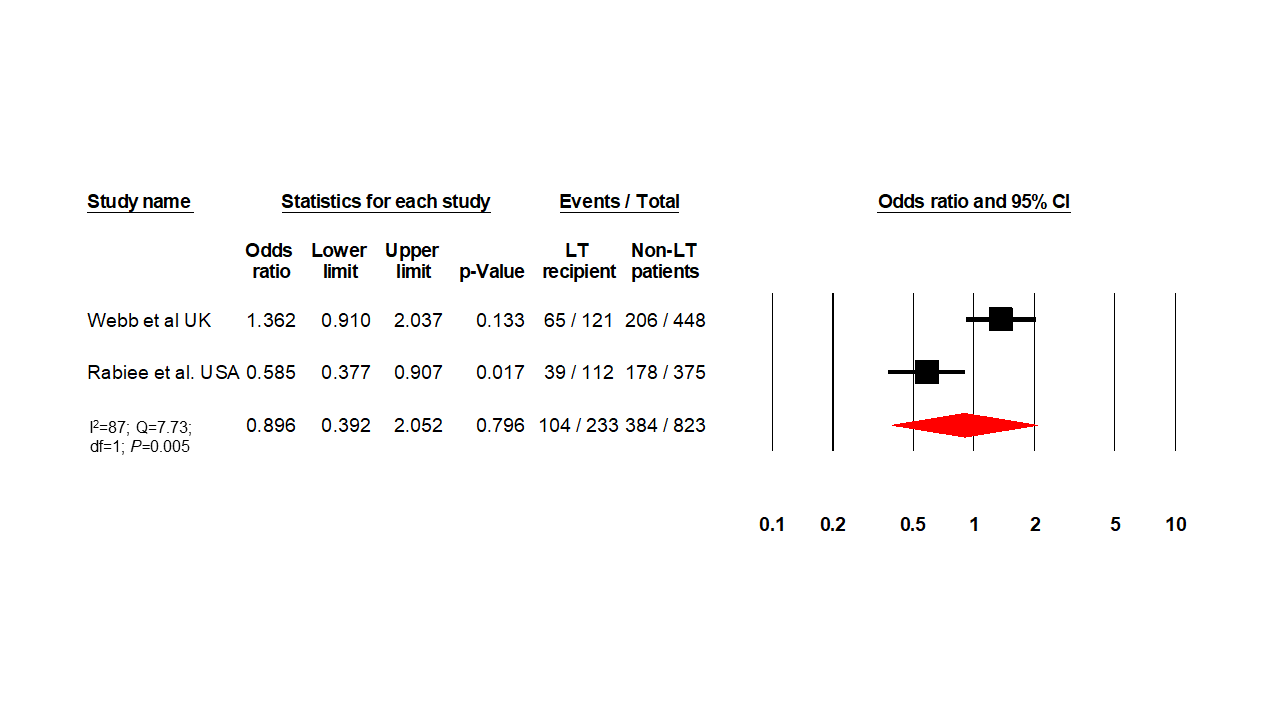


**Supplementary figure 7: Cumulative percentage of patients with change in immunosuppression post COVID-19 infection.**


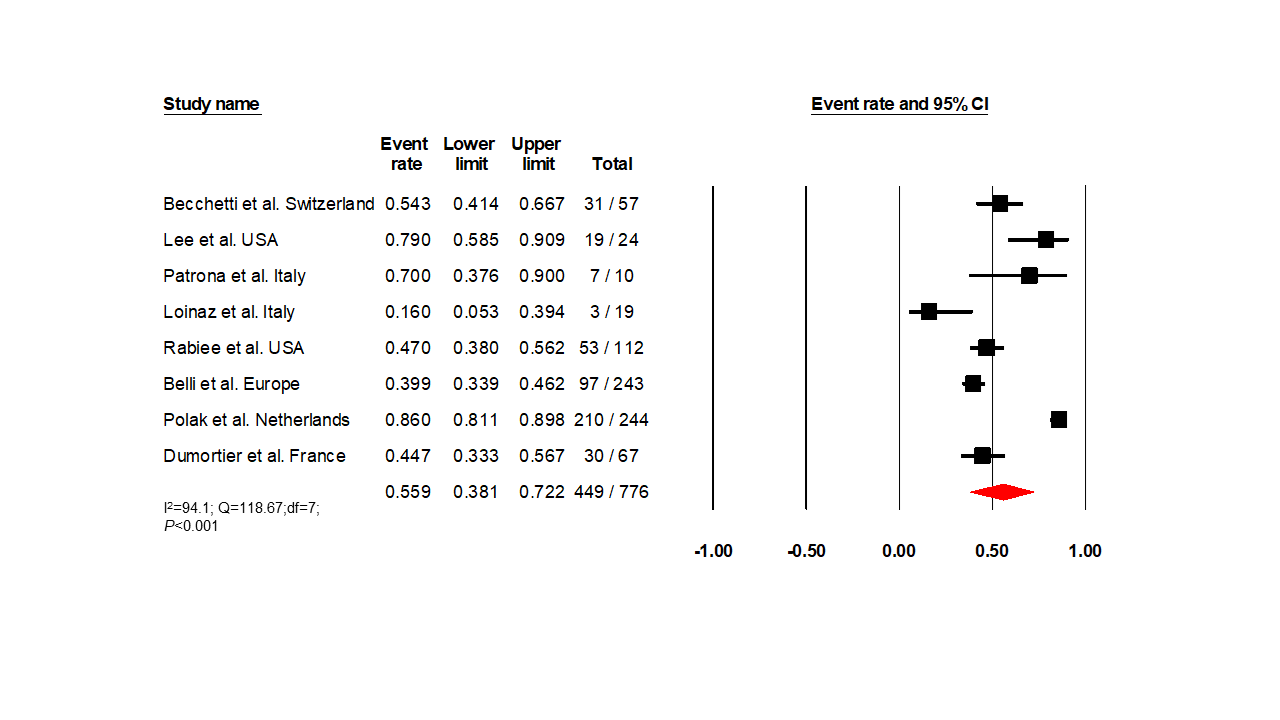


**Supplementary figure 8: Funnel plot of the studies included for incidence of a) hospitalization, b) intensive care unit admission, c) elevated liver chemistries, and d) ventilatory support.**


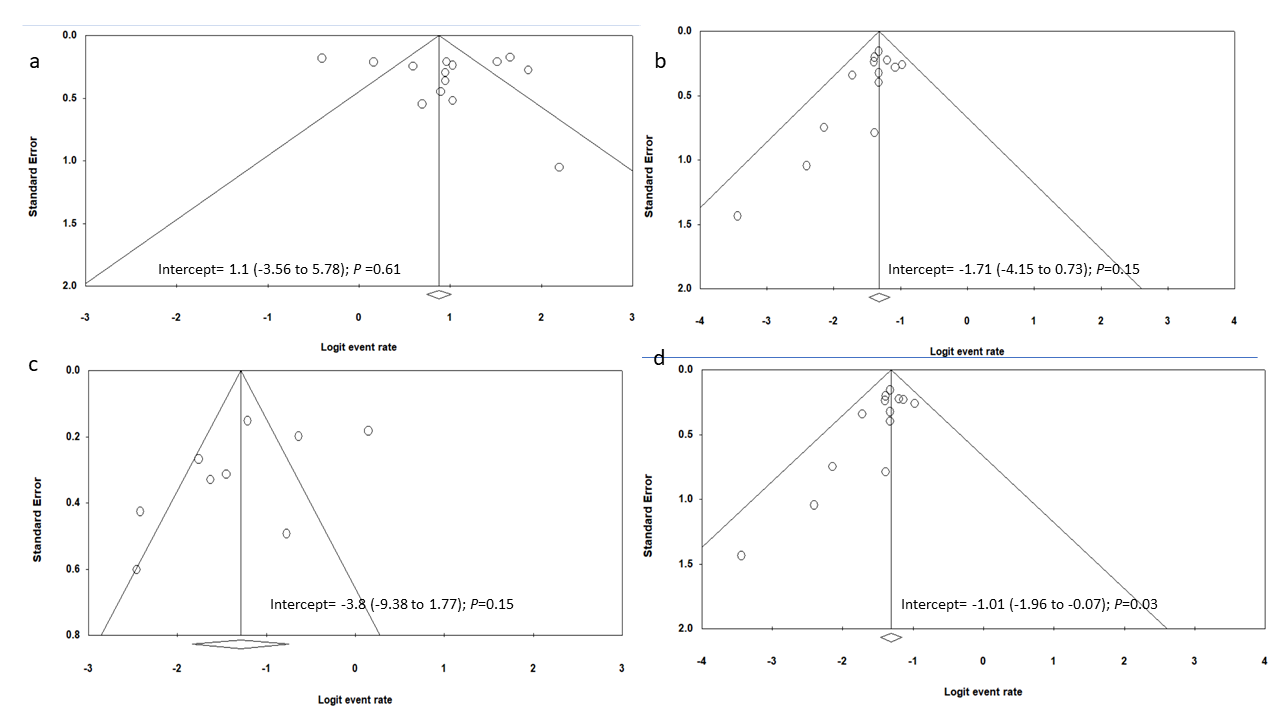


Appendix I

((COVID-19[MeSH Terms]) AND (liver transplantation[MeSH Terms])) AND (("2019/12/01"[Date - Entry] : "2021/05/20"[Date - Entry]))-158

((SARS-CoV-2[MeSH Terms]) AND (liver transplantation[MeSH Terms])) AND (("2019/12/01"[Date - MeSH] : "2021/05/20"[Date - MeSH]))-130

Appendix II: Diagnosis of COVID-19

| **Serial no** | **First author, Country, Centres. ^Reference no.^** | **Reason for testing** | **COVID-19 diagnosis** | **Severity definition** |
| --- | --- | --- | --- | --- |
| 1 | Colmenero et al., Spain, Multicentre. ^22^ | Symptomatic. Asymptomatic patients were tested for COVID-19 because of incidental radiological findings (n = 2) or after a high-risk contact (n = 5). | PCR + | Need for mechanical ventilation, admission to intensive care unit, and/or death. |
| 2 | Becchetti et al., Switzerland, Multicentre. ^17^ | Symptomatic | PCR + | Requiring mechanical ventilation |
| 3 | Lee et al., USA, Single centre. ^23^ | Symptomatic (one patient each tested + prior to endoscopy and hospital admission as a part of screening tests) | PCR + | Requiring advanced oxygen device delivery use |
| 4 | Webb et al., UK, Multicentre. ^24^ | Symptomatic patients presenting to hospital | PCR + | Requiring invasive ventilation |
| 5 | Malekhosseini. et al  Iran, Single centre. ^25^ | Symptomatic | All the patients had either chest CT scan or PCR confirmation for diagnosis of COVID-19 | SpO2 <90% |
| 6 | Patrono et al., Italy,  Single centre. ^26^ | Symptomatic | PCR/CT/Symptoms | Non-invasive ventilation |
| 7 | Loinaz et al., Italy,  Single centre. ^27^ | Symptomatic | PCR + (only 2 had classical symptoms of COVID-19 but negative PCR) | Need for mechanical ventilation and/or death. |
| 8 | Dhampalwar et al.,  India, Single centre.^28^ | Symptomatic (one turned positive on screening due to history of contact) | PCR + | Patient who expired |
| 9 | Rabiee et al.,  USA, Multicentre. ^29^ | Not reported (mostly symptomatic) | PCR + | Admission to the intensive care unit, receipt of vasopressors, or mechanical ventilation |
| 10 | Mansoor et al., USA, Multicentre. ^16^ | Symptomatic | PCR + |  |
| 11 | Belli et al., Europe, Multicentre. ^30^ | Symptomatic | PCR + | Ventilatory support |
| 12 | Trapani et al., Italy, Multicentre.^31^ | Symptomatic | PCR + | Not defined (Mentions severe clinical state) |
| 13 | Polak et al. Netherlands, Multicentre. ^32^ | Symptomatic (survey-based study) | PCR + and/or highly suggestive lung injury on thoracic CT scan | Mortality due to COVID-19 |
| 14 | Gruttadauria et al., Italy, Multicentre. ^33^ | Unclear (? Symptomatic) Survey-based study | PCR + | Mortality due to COVID-19 |
| 15 | Pereira et al., USA, Multicentre.^34^ | Symptomatic | PCR+ | Mechanical ventilation, admission to intensive care unit or death |
| 16 | Ali et al., Saudi Arabia, Single centre. ^35^ | Symptomatic. 17 asymptomatic patients detected to be PCR + as a part of screening for hospital admission/prior to procedure/contact exposure. | PCR+ | Chinese National Health Commission criteria |
| 17 | Kates et al., USA, Multicentre. ^36^ | Unclear | PCR+ | Need of ventilatory support |
| 18 | Dumortier et al. France, Multicentre.^37^ | Symptomatic | PCR+ or Symptoms+, CT+ and COVID-19 antibodies. | Need for mechanical ventilation, admission to intensive care unit, or death. |

PCR-polymerase chain reaction.
