# Supplementary material for: Impact of COVID-19 on liver transplant recipients–A systematic review and meta-analysis
Source: eClinicalMedicine. 2021 Jul 13;38:101025. doi: 10.1016/j.eclinm.2021.101025 (PMC8276632; doi:10.1016/j.eclinm.2021.101025)
Supplement: Supplementary file 2 [file mmc2.docx]

**Supplementary file 2: Excluded case reports and other language articles**

| Reference No. | First author, Year | N | Age | Gender | Indication for LT | Time to COVID-19 infection | Diagnosis of COVID-19 infection (PCR/CT/Symptoms) | Severity of infection | Immunosuppression continued (Yes or No) | If changed what was changed in immunosuppression | Alive/Dead | Unique conclusion by authors |
| --- | --- | --- | --- | --- | --- | --- | --- | --- | --- | --- | --- | --- |
| 1 | Huang et al;2020 | 1 | 59 | Male | CLD-HBV  HCC | 3 years | PCR | Day-1: Mild  Day-4: Severe | Yes | Dose of Tac and MMF halved | Dead |  |
| 2 | Qin et al;2020 | 1 | 37 | Male | CLD-HBV  HCC | 9 days | PCR | - | Yes | Steroids and Tac reduced to minimum dose | Alive | strict screening guidance for organ recipients and donors with incubated COVID-19 needs to be developed |
| 3 | Liu et al;2020 | 1 | 50 | Male | CLD-HBV | 2.5 years | PCR | Severe | Yes | Tac discontinued, methylprednisolone started | Alive | Temporary withdrawal of immunosuppression and administration of corticosteroid in low-dose might be principle components of therapeutic regime |
| 4 | Kates et al,2020 | 1 | 67 | Male | CLD-HCV | 19 years | PCR | mild | No (same dose of cyclosporine) |  | Alive | The practice of routinely reducing immunosuppression in the context of viral illness may need to be re-examined |
| 5 | Nikoupour et al,2020 | 1 | 3year | Male | Biliary atresia | 1·5 year | PCR | Severe | Yes | Discontinued after 2 days. | Dead |  |
| 6 | Gao et al,2020 | 3 | 59 years | Male | HCC | 2·75 years | PCR |  | Yes | Tac/MMF same dose  Steroid added | Dead | Reduction or temporary halt to immunosuppressive agents might be considered in patients with serious infections. |
|  |  |  | 50 years | Male | CLD-HBV | 2·5 years | PCR |  | Yes | Tac stopped. Steroid added | Alive |  |
|  |  |  | 37 year | Male | HCC | 3d before LT | PCR |  | Yes | Tac/steroid dose reduced | Alive |  |
| 7 | Muller et al, 2020 | 1 | 55year | Male | CLD-HCV with HCC | 15 months | PCR | moderate | No |  | Alive | HIV co-infected |
| 8 | Morand et al, 2020 | 1 | 55months | Female | Biliary atresia | 5 months | PCR | mild | Yes | Tac continued  Dose reduced to half | Alive | Patient had co-infection with EBV also |
| 9 | Hammami et al, 2020 | 1 | 63 year | Male | CLD-HCV with HCC | 10 years | PCR | mild | No |  | Not given | Patient was having ESRD on MHD |
| 10 | Verma et al, 2020 | 5 | 36 years | Male | FAP | 9 years | PCR |  | No |  | Alive |  |
|  |  |  | 54 years | Male | Chronic BCS | 16 years | PCR |  | Yes | MMF stopped, Prednisolone doubled | Not given |  |
|  |  |  | 54 years | Male | PSC | 6 years | PCR |  | No |  | Alive |  |
|  |  |  | 23 years | Male | ARPKD+CHF | 1·5 years | PCR |  | No |  | Alive |  |
|  |  |  | 28years | Male | PFIC-3 | 1year | PCR |  | No |  | Not given |  |
| 11 | Fishman et al;2020 | 1 | 66 year | Male | ESLD-ethanol +HBV HCC | 22months | PCR | severe | Yes | MMF reduced to half  Tac continued  Steroid started | Alive |  |
| 12 | Nikoupour et al;2020 | 1 | 35years | Male | PSC with UC | 3 years | PCR | mild | Yes | MMF reduced  Tac/steroid continued | Alive | patients who are receiving immunosuppressive medication may not be at higher risks of developing severe symptoms related to COVID |
| 13 | Modi et al;2020 | 1 | 32 year | Male | Drug induced ALF | 7 year | PCR | moderate | Yes | MMF stopped  Tac reduced  Steroid continued | Alive | HIV co-infected |
| 14 | Sessa et al;2020 | 1 | 58 year | Male | CLD-HBV  HCC | 18months | PCR | Mild | No |  | Alive |  |
| 15 | Niess et al;2020 | 1 | 56year | Male | CLD-HBV | 10 days | PCR | asymptomatic | No |  | Alive | Patient had COVID pre LT, Documented negative at time of LT;  Repeat RT PCR positive after LT |
| 16 | Heinz et al;2020 | 1 | 6 months | Female | Biliary atresia | 4 days | PCR | severe | Yes | MMF stopped but due to diarrhea | Not Given | Associated Biopsy proven ACR |
| 17 | Waisberg et al, 2020 | 5 | 69 year | Female | CLD-HCV with HCC | 9days | PCR | Severe | Yes | IS stopped | Dead | Multiple co-morbidities |
|  |  |  | 67 years | Male | CLD-NASH | 36 days | PCR | Severe | Yes | MMF stopped.  Tac reduced. | Dead | CMV co-infection |
|  |  |  | 69 years | Male | Alcohol related cirrhosis | 10 days | PCR | Mild | No |  | Alive |  |
|  |  |  | 59 years | Male | Cryptogenic cirrhosis | 11 days | PCR | Mild | Yes | MMF stopped Tac same dose | Alive |  |
|  |  |  | 34 years | Male | PSC | 18 days | PCR | Mild | No |  | Alive |  |
| 18 | Mehta et al, 2020 | 3 | 68 years | Female | Not mentioned | 353 days | PCR | Mild | No |  | Alive | Donor of all patients were HIV+ve |
|  |  |  | 65 years | Male | Not mentioned | 77 days | PCR | Mild | Yes | Tac and MMF stopped | Alive |  |
|  |  |  | 62 year | Male | Not mentioned | 287 days | PCR | Mild | No |  | Alive |  |
| 19 | De Gottardi et al, 2020 | 1 | 62 years | Male | CLD-HCV/NASH with  HCC | 4 years | RT PCR | Moderate to severe | Yes | Sirolimus reduced to half.  No mention of MMF dosage | Alive |  |
| 20 | Ossami Saidy et al;2020 | 3 | 45 years | Male | CLD with HCC | 7 months | PCR | Asymptomatic | No |  | Alive |  |
|  |  |  | 77 years | Female | PBC | 21 years | PCR | Asymptomatic | No |  | Alive |  |
|  |  |  | 70 years | Male | CLD with HCC | 12 years | PCR | Asymptomatic | Patient not on IS |  | Alive |  |
| 21 | García-Juárez et al;2020 (Article in Spanish) | 1 | 64 years | Female | HCV cirrhosis | ? 2 years | PCR· | - | Yes | Tacrolimus reduced | Alive | Even though tacrolimus was continued (low dose) the patient improved. |
| 22 | Mathiasen et al;2020 | 1 | 58 years | Female | BCS | 2 years | PCR | Mild-moderate | No |  | Alive |  |
| 23 | Dajti et al;2020 | 1 | 27 year | Male | PSC with UC | 2 years | Not mentioned | Mild | Yes | Stopped in view of high Tac level caused by drug interaction | Alive | Attempt to continue IS but stopped in view of drug-drug interaction. |
| 24 | Terrabuio et al;2020 | 4 | 42 years | Female | CLD-HCV | 5 years | PCR | Not mentioned | Unknown (NR) |  | Alive |  |
|  |  |  | 59 years | Female | CLD-NASH | 3 years | PCR | Not mentioned | Unknown (NR) |  | Alive |  |
|  |  |  | 62 years | Female | ALF | 8 years | PCR | Not mentioned | Unknown (NR) |  | Alive |  |
|  |  |  | 43 year | Male | PSC | 8 years | PCR | Not mentioned | Unknown (NR) |  | Alive |  |
| 25 | Kolonko et al;2020 | 1 | 52 | Male | PSC with UC | 8 days | Not mentioned | Mild | Yes | MMF stopped  TAC and prednisolone continued. | Alive |  |
| 26 | Merli et al;2020 | 1 | 50 years | Female | Secondary sclerosing cholangitis | 41 days | PCR | Mild | No |  | Alive |  |
| 27 | Zhong et al. 2020 | 1 | 37 years | Male | HBV with HCC | 9 days | PCR |  | Yes | Tac stopped.  Methylprednisolone started | Alive | Developed acute rejection |
| 28 | Bhoori et al, 2020 | 3 +3 |  | Male | - | - | PCR | - | - | - | Dead -3  Alive-3 | Increased risk in long term transplant recipients |
| 29 | Mocchegiani et al, 2020 | 3 | - | - |  | - | - | Mild | - | - | Alive |  |
| 30 | Ringer et al. 2021 | 3 | - | - | - | - | - | - | - | - | - | - |
| 31 | Tannur et al. 2020 | 1 | Child |  |  |  | PCR |  |  |  | Alive | Only one out of 845 LT children developed mild infected. |
| 32 | Choudhury et al. 2020 | 6 | 52 years | Male | CLD-NASH | 16 months | PCR | Severe | Yes | Tac and MMF stopped, Dexamethasone started | Alive |  |
|  |  |  | 39 years | Male | CLD-Ethanol | 72 months | PCR | Moderate | Yes | Tac and MMF stopped; prednisolone started | Alive |  |
|  |  |  | 48 years | Male | CLD-NASH | 72 months | PCR | Mild | Yes | Tac stopped, prednisolone started | Alive |  |
|  |  |  | 50 years | Male | CLD-Ethanol | 17 months | PCR | Mild | Yes | Tac stopped, prednisolone started | Alive |  |
|  |  |  | 38 years | Male | CLD-Ethanol | 60 months | PCR | Mild | Yes | Not mentioned | Alive |  |
|  |  |  | 48 years | Male | CLD-Ethanol | 24 months | PCR | Asymptomatic | Yes | Everolimus stopped; prednisolone started | Alive |  |
| 33 | De Souza, 2020 | 1 | 67 | Female | ?alpha-1 antitrypsin deficieny | - | - | Moderate | - | - | Alive |  |
| 34 | Buscemi et al. 2020 | 1 | 61 | Male | HCV cirrhosis with HCC | 9 days | PCR | Mild | No | Continued | Alive |  |
| 35 | Lagana et al. 2020 | 1 | 6 months | Female | Biliary atresia | 4 days | PCR | Severe | - | - | Alive |  |
| 36 | Fernandez-Ruiz et al. 2020 | 6 | 63 | Male | HBV with HCV cirrhosis with HCC |  | PCR | Severe | - | - | Alive |  |
|  |  |  | 72 | Male | Cryptogenic cirrhosis |  | PCR | Mild | - | - | Alive |  |
|  |  |  | 79 | Female | HCV with HCC |  | PCR | Severe | - | - | Dead |  |
|  |  |  | 73 | Male | HBV cirrhosis |  | PCR | Severe | - | - | Dead |  |
|  |  |  | 76 | Female | HCV cirrhosis |  | PCR | Mild | - | - | Alive |  |
|  |  |  | 46 | Female | ALF |  | PCR | Mild | - | - | Alive |  |
| 37 | Bhatti et al. 2020 | Not accessible | | | | | | | | | | |
| 38 | Rodríguez-Gandía et al, 2020 | Article in Spanish (case report of one fulminant liver failure patients undergoing liver transplant during COVID-19 pandemic) | | | | | | | | | | |
| 39 | Gondolesi et al, 2020 | Article in Spanish (Impact of COVID-19 on transplant programmes) | | | | | | | | | | |
| 40 | Remport et al. 2020 | Article in Hungarian | | | | | | | | | | |
|  | Total | 74 patients (57 alive, 10 died and 7 unknown) | | | Immunosuppression changed in 29 patients of which five patients died and 21 were alive and 2 not known.  Immunosuppression continued at same dose in 19 patients of which 17 were alive and 2 not known.  Immunosuppression status not known in 21 patients of which 13 were alive, 3 unknown and 5 died.  Not on immnosuppression-1 (alive) | | | | | | | |

LT=Liver Transplantation, IS=Immunosuppression, RT-PCR= Reverse Transcriptase Polymerase Chain reaction, CT=Computerised Tomography, CLD=Chronic Liver disease, HCV=Hepatitis C Virus, HBV=hepatitis B Virus, PSC=Primary sclerosing Cholangitis, UC=ulcerative colitis, HCC=hepatocellular carcinoma, ALF=acute liver failure, NASH=Non-alcoholic steatohepatitis, PBC=primary biliary cirrhosis, BCS=Budd Chiari syndrome, Tac=tacrolimus, MMF=mycophenolate mofetil, NR-not reported.

**References:**

1. Huang JF, Zheng KI, George J, Gao HN, Wei RN, Yan HD, Zheng MH. Fatal outcome in a liver transplant recipient with COVID-19. Am J Transplant. 2020 Jul;20(7):1907-1910. doi: 10.1111/ajt.15909. Epub 2020 May 4. PMID: 32277591; PMCID: PMC7262021.
2. Qin J, Wang H, Qin X, Zhang P, Zhu L, Cai J, Yuan Y, Li H. Perioperative Presentation of COVID-19 Disease in a Liver Transplant Recipient. Hepatology. 2020 Oct;72(4):1491-1493. doi: 10.1002/hep.31257. PMID: 32220017.
3. Liu B, Wang Y, Zhao Y, Shi H, Zeng F, Chen Z. Successful treatment of severe COVID-19 pneumonia in a liver transplant recipient. Am J Transplant. 2020 Jul;20(7):1891-1895. doi: 10.1111/ajt.15901. Epub 2020 Apr 19. PMID: 32243673.
4. Kates OS, Fisher CE, Stankiewicz-Karita HC, Shepherd AK, Church EC, Kapnadak SG, Lease ED, Riedo FX, Rakita RM, Limaye AP. Earliest cases of coronavirus disease 2019 (COVID-19) identified in solid organ transplant recipients in the United States. Am J Transplant. 2020 Jul;20(7):1885-1890. doi: 10.1111/ajt.15944. Epub 2020 May 8. PMID: 32330356; PMCID: PMC7264737.
5. Nikoupour H, Kazemi K, Arasteh P, Ghazimoghadam S, Eghlimi H, Dara N, Gholami S, Nikeghbalian S. Pediatric liver transplantation and COVID-19: a case report. BMC Surg. 2020 Oct 6;20(1):224. doi: 10.1186/s12893-020-00878-6. PMID: 33023552; PMCID: PMC7538038.
6. Gao F, Zheng KI, Gu JY, George J, Zheng MH. COVID-19 and liver transplantation: Lessons learned from three reported cases. Transpl Infect Dis. 2020 Aug;22(4):e13335. doi: 10.1111/tid.13335. Epub 2020 Jun 2. PMID: 32438464; PMCID: PMC7267077.
7. Müller H, Kniepeiss D, Stauber R, Schrem H, Rauter M, Krause R, Schemmer P. Recovery from COVID-19 following hepatitis C, human immunodeficiency virus infection, and liver transplantation. Am J Transplant. 2020 Nov;20(11):3255-3256. doi: 10.1111/ajt.16107. Epub 2020 Jun 18. PMID: 32492753; PMCID: PMC7300671.
8. Morand A, Roquelaure B, Colson P, Amrane S, Bosdure E, Raoult D, Lagier JC, Fabre A. Child with liver transplant recovers from COVID-19 infection. A case report. Arch Pediatr. 2020 Jul;27(5):275-276. doi: 10.1016/j.arcped.2020.05.004. Epub 2020 May 6. PMID: 32402433; PMCID: PMC7200359.
9. Hammami MB, Garibaldi B, Shah P, Liu G, Jain T, Chen PH, Kim AK, Avdic E, Petty B, Strout S, Fine DM, Niranjan-Azadi A, Garneau WM, Cameron AM, Monroy Trujillo JM, Gurakar A, Avery R. Clinical course of COVID-19 in a liver transplant recipient on hemodialysis and response to tocilizumab therapy: A case report. Am J Transplant. 2020 Aug;20(8):2254-2259. doi: 10.1111/ajt.15985. Epub 2020 Jun 3. PMID: 32359210; PMCID: PMC7267667.
10. Verma A, Khorsandi SE, Dolcet A, Prachalias A, Suddle A, Heaton N, Jassem W. Low prevalence and disease severity of COVID-19 in post-liver transplant recipients-A single centre experience. Liver Int. 2020 Aug;40(8):1972-1976. doi: 10.1111/liv.14552. Epub 2020 Jun 17. PMID: 32471013; PMCID: PMC7300707.
11. Fishman JA, Roberts MB, Zhang EW, Kumar D, Hirsch HH, Maggiore U. Case 29-2020: A 66-Year-Old Man with Fever and Shortness of Breath after Liver Transplantation. N Engl J Med. 2020 Sep 17;383(12):1168-1180. doi: 10.1056/NEJMcpc2004982. PMID: 32937051; PMCID: PMC7510944.
12. Nikoupour H, Arasteh P, Gholami S, Nikeghbalian S. Liver transplantation and COVID-19: a case report and cross comparison between two identical twins with COVID-19. BMC Surg. 2020 Aug 8;20(1):181. doi: 10.1186/s12893-020-00837-1. PMID: 32770973; PMCID: PMC7414266.
13. Modi AR, Koval CE, Taege AJ, Modaresi Esfeh J, Eghtesad B, Narayanan Menon KV, Quintini C, Miller C. Coronavirus disease 2019 in an orthotopic liver transplant recipient living with human immunodeficiency virus. Transpl Infect Dis. 2020 Oct;22(5):e13351. doi: 10.1111/tid.13351. Epub 2020 Jun 17. PMID: 32500666; PMCID: PMC7300493.
14. Sessa A, Mazzola A, Lim C, Atif M, Pappatella J, Pourcher V, Scatton O, Conti F. COVID-19 in a liver transplant recipient: Could iatrogenic immunosuppression have prevented severe pneumonia? A case report. World J Gastroenterol. 2020 Nov 28;26(44):7076-7084. doi: 10.3748/wjg.v26.i44.7076. PMID: 33311951; PMCID: PMC7701943.
15. Niess H, Börner N, Muenchhoff M, Khatamzas E, Stangl M, Graf A, Girl P, Georgi E, Koliogiannis D, Denk G, Irlbeck M, Werner J, Guba M. Liver transplantation in a patient after COVID-19 - Rapid loss of antibodies and prolonged viral RNA shedding. Am J Transplant. 2021 Apr;21(4):1629-1632. doi: 10.1111/ajt.16349. Epub 2020 Nov 2. PMID: 33047475; PMCID: PMC7675727.
16. Heinz N, Griesemer A, Kinney J, Vittorio J, Lagana SM, Goldner D, Velasco M, Kato T, Lobritto S, Martinez M. A case of an Infant with SARS-CoV-2 hepatitis early after liver transplantation. Pediatr Transplant. 2020 Dec;24(8):e13778. doi: 10.1111/petr.13778. Epub 2020 Jun 25. PMID: 32559354; PMCID: PMC7323125.
17. Waisberg DR, Abdala E, Nacif LS, Haddad LB, Ducatti L, Santos VR, Gouveia LN, Lazari CS, Martino RB, Pinheiro RS, Arantes RM, Terrabuio DR, Malbouisson LM, Galvao FH, Andraus W, Carneiro-D'Albuquerque LA. Liver transplant recipients infected with SARS-CoV-2 in the early postoperative period: Lessons from a single center in the epicenter of the pandemic. Transpl Infect Dis. 2021 Feb;23(1):e13418. doi: 10.1111/tid.13418. Epub 2020 Aug 4. PMID: 32667716; PMCID: PMC7404440.
18. Mehta SA, Rana MM, Motter JD, Small CB, Pereira MR, Stosor V, Elias N, Haydel B, Florman S, Odim J, Morsheimer M, Robien M, Massie AB, Brown D, Boyarsky BJ, Garonzik-Wang J, Tobian AAR, Werbel WA, Segev DL, Durand CM; HOPE in Action Investigators. Incidence and Outcomes of COVID-19 in Kidney and Liver Transplant Recipients With HIV: Report From the National HOPE in Action Consortium. Transplantation. 2021 Jan 1;105(1):216-224. doi: 10.1097/TP.0000000000003527. PMID: 33165238; PMCID: PMC8018537.
19. De Gottardi A, Fratila C, Bertoli R, Cerny A, Magenta L, Gianella P, Majno-Hurst P, Ceschi A, Vanini G, Bernasconi E. Clinical characteristics and management of a liver transplanted patient admitted with SARS-CoV-2 infection. Clin Res Hepatol Gastroenterol. 2020 Nov;44(6):e141-e144. doi: 10.1016/j.clinre.2020.05.014. Epub 2020 Jun 10. PMID: 32565199; PMCID: PMC7284277.
20. Ossami Saidy RR, Globke B, Pratschke J, Schoening W, Eurich D. Successful implementation of preventive measures leads to low relevance of SARS-CoV-2 in liver transplant patients: Observations from a German outpatient department. Transpl Infect Dis. 2020 Dec;22(6):e13363. doi: 10.1111/tid.13363. Epub 2020 Jun 22. PMID: 32516847; PMCID: PMC7300929.
21. García-Juárez I, Campos-Murguía A, Tovar-Méndez VH, Gabutti A, Ruiz I. Unexpected better outcome in a liver transplant recipient with COVID-19: a beneficial effect of tacrolimus? Rev Gastroenterol Mex. 2020 Oct-Dec;85(4):437-442. English, Spanish. doi: 10.1016/j.rgmx.2020.08.001. Epub 2020 Sep 16. PMID: 33032841; PMCID: PMC7494268.
22. Mathiasen VD, Oversoe SK, Ott P, Jensen-Fangel S, Leth S. Recovery of Moderate Coronavirus Disease 2019 in a Liver Transplant Recipient on Continued Immunosuppression: A Case Report. Transplant Proc. 2020 Nov;52(9):2703-2706. doi: 10.1016/j.transproceed.2020.09.007. Epub 2020 Sep 17. PMID: 33039144; PMCID: PMC7498236.
23. Dajti E, Cristini F, Tamanini G, Cescon M, Bazzoli F, Tamè M. COVID-19 in a young liver transplant recipient: caution for drug-drug interactions. J Gastrointestin Liver Dis. 2020 Sep 9;29(3):470. doi: 10.15403/jgld-2672. PMID: 32830825.
24. Terrabuio DRB, Haddad L, Ducatti L, Nunes Gouveia L, Rocha-Santos V, Ferreira RMT, Darce GF, Cardoso AJA, Carrilho FJ, Andraus W, Abdala E, D Albuquerque LAC. Insights in the approach of long-term liver transplant recipients with COVID-19. Transpl Infect Dis. 2021 Feb;23(1):e13424. doi: 10.1111/tid.13424. Epub 2020 Aug 8. PMID: 32702167; PMCID: PMC7404373.
25. Kolonko A, Dudzicz S, Wiecek A, Król R. COVID-19 infection in solid organ transplant recipients: A single-center experience with patients immediately after transplantation. Transpl Infect Dis. 2021 Feb;23(1):e13381. doi: 10.1111/tid.13381. Epub 2020 Jul 6. PMID: 32578289; PMCID: PMC7361238.
26. Merli M, Alteri C, Colagrossi L, Perricone G, Chiappetta S, Travi G, Campisi D, Pugliano MT, Vecchi M, Orcese C, Rossini S, De Carlis L, Vismara C, Belli L, Perno CF, Puoti M. Mild Course of SARS-CoV-2 Infection in a Liver Transplant Recipient Undergoing Plasma Exchange and Defibrotide for Acute Graft Rejection. Transplantation. 2021 Feb 1;105(2):e22-e24. doi: 10.1097/TP.0000000000003592. PMID: 33492115.
27. Zhong Z, Zhang Q, Xia H, Wang A, Liang W, Zhou W, Zhou L, Liu X, Rao L, Li Z, Peng Z, Mo P, Xiong Y, Ye S, Wang Y, Ye Q. Clinical characteristics and immunosuppressant management of coronavirus disease 2019 in solid organ transplant recipients. Am J Transplant. 2020 Jul;20(7):1916-1921. doi: 10.1111/ajt.15928. Epub 2020 May 4. PMID: 32282986; PMCID: PMC7262295.
28. Bhoori S, Rossi RE, Citterio D, Mazzaferro V. COVID-19 in long-term liver transplant patients: preliminary experience from an Italian transplant centre in Lombardy. Lancet Gastroenterol Hepatol. 2020 Jun;5(6):532-533. doi: 10.1016/S2468-1253(20)30116-3. Epub 2020 Apr 9. PMID: 32278366; PMCID: PMC7146678.
29. Mocchegiani F, Baroni GS, Vivarelli M. Mild impact of SARS-CoV-2 infection on the entire population of liver transplant recipients: the experience of an Italian Centre based in a high-risk area. Updates Surg. 2020 Dec;72(4):1291-1293. doi: 10.1007/s13304-020-00881-9. Epub 2020 Sep 10. PMID: 32914336; PMCID: PMC7483055.
30. Ringer M, Azmy V, Kaman K, Tang D, Cheung H, Azar MM, Price C, Malinis M. A retrospective matched cohort single-center study evaluating outcomes of COVID-19 and the impact of immunomodulation on COVID-19-related cytokine release syndrome in solid organ transplant recipients. Transpl Infect Dis. 2021 Apr;23(2):e13556. doi: 10.1111/tid.13556. Epub 2021 Jan 22. PMID: 33378571; PMCID: PMC7883059.
31. Tannuri U, Tannuri ACA, Cordon MNA, Miyatani HT. Low incidence of COVID-19 in children and adolescent post-liver transplant at a Latin American reference center. Clinics (Sao Paulo). 2020 Jun 3;75:e1986. doi: 10.6061/clinics/2020/e1986. PMID: 32520226; PMCID: PMC7247737.
32. Choudhury A, Reddy GS, Venishetty S, Pamecha V, Shasthry SM, Tomar A, Mitra LG, Prasad VST, Mathur RP, Bhattacharya D, Sarin SK. COVID-19 in Liver Transplant Recipients - A Series with Successful Recovery. J Clin Transl Hepatol. 2020 Dec 28;8(4):467-473. doi: 10.14218/JCTH.2020.00061. Epub 2020 Oct 10. PMID: 33447532; PMCID: PMC7782113.
33. De Souza L, Nwanji V, Kaur G. An auspicious triumph of recovery from dialysis-requiring acute kidney injury in COVID-19 in a patient with chronic kidney disease, α-1 antitrypsin deficiency, and liver transplant: A case report. Clin Nephrol. 2020 Dec;94(6):297-306. doi: 10.5414/CN110294. PMID: 32909541.
34. Buscemi V, De Carlis R, Lauterio A, Merli M, Puoti M, De Carlis L. Does interval time between liver transplant and COVID-19 infection make the difference? Dig Liver Dis. 2021 Feb;53(2):169-170. doi: 10.1016/j.dld.2020.08.027. Epub 2020 Aug 25. PMID: 32921600; PMCID: PMC7447264.
35. Lagana SM, De Michele S, Lee MJ, Emond JC, Griesemer AD, Tulin-Silver SA, Verna EC, Martinez M, Lefkowitch JH. COVID-19 Associated Hepatitis Complicating Recent Living Donor Liver Transplantation. Arch Pathol Lab Med. 2020 Apr 17. doi: 10.5858/arpa.2020-0186-SA. Epub ahead of print. PMID: 32302212.
36. Fernández-Ruiz M, Andrés A, Loinaz C, Delgado JF, López-Medrano F, San Juan R, González E, Polanco N, Folgueira MD, Lalueza A, Lumbreras C, Aguado JM. COVID-19 in solid organ transplant recipients: A single-center case series from Spain. Am J Transplant. 2020 Jul;20(7):1849-1858. doi: 10.1111/ajt.15929. Epub 2020 May 10. PMID: 32301155.
37. Bhatti ABH, Riyaz S, Akhtar A. In-hospital Mortality after Liver Transplantation due to COVID-19. J Coll Physicians Surg Pak. 2020 Oct;30(10):141-142. doi: 10.29271/jcpsp.2020.supp2.141. PMID: 33115594.
38. Rodríguez-Gandía MA, López-Hervás P, Téllez L, Gajate L; en nombre de la Unidad de Trasplante Hepático. Successful urgent liver transplant due to fulminant autoimmune hepatitis during the height of the COVID-19 pandemic in Spain. Gastroenterol Hepatol. 2020 Nov;43(9):537-538. English, Spanish. doi: 10.1016/j.gastrohep.2020.06.002. Epub 2020 Jun 11. PMID: 32651042; PMCID: PMC7287481.
39. Gondolesi GE, Reyes-Toso ML, Bisigniano L, de Santibañes M, Pekolj J, Maurette R, Quiñonez EG, Maraschio MA, Imventarza O, Lendoire J, Bitetti L, Ruf A, Aballay G, Gil O, Mattera FJ, Barros Schelotto P, Descalzi VI. Impacto de la pandemia por COVID-19 sobre el trasplante hepático en la Argentina. Otro daño colateral [Impact of COVID-19 pandemic in liver transplantation in Argentina. Other collateral damage]. Medicina (B Aires). 2020;80 Suppl 6:71-82. Spanish. PMID: 33481736.
40. Remport Á, Gerlei Z, Cseprekál O, Wagner L, Földes K, Marton A, Patonai A, Török S, Haboub-Sandil A, Varga M, Doros A, Smudla A, Fazakas J, Kóbori L. Az új koronavírus (SARS-CoV-2) okozta fertőzésben szenvedő vese- és májátültetett betegek ellátásának speciális szempontjai. (A COVID-19-pandémia orvosszakmai kérdései) [Guidance on the special care of liver or kidney transplant recipients diagnosed with COVID-19]. Orv Hetil. 2020 Aug;161(32):1310-1321. Hungarian. doi: 10.1556/650.2020.31923. PMID: 32750019.
