# Supplementary material for: Impact of COVID-19 on liver transplant recipients–A systematic review and meta-analysis
Source: eClinicalMedicine. 2021 Jul 13;38:101025. doi: 10.1016/j.eclinm.2021.101025 (PMC8276632; doi:10.1016/j.eclinm.2021.101025)
Supplement: Supplementary file 3 [file mmc3.docx]

**Caption for supplementary material**

Supplementary file 1

Supplementary file 2 (Excluded case reports)

Supplementary file 3 (MOOSE checklist)
